# Fracture biomechanics influence local and systemic immune responses in a murine fracture-related infection model

**DOI:** 10.1242/bio.057315

**Published:** 2021-10-01

**Authors:** Marina Sabaté-Brescó, Corina M. Berset, Stephan Zeiter, Barbara Stanic, Keith Thompson, Mario Ziegler, R. Geoff Richards, Liam O'Mahony, T. Fintan Moriarty

**Affiliations:** 1 AO Research Institute Davos, AO Foundation, 7270, Davos, Switzerland; 2 Swiss Institute of Asthma and Allergy Research, University of Zurich, 7270, Davos, Switzerland

**Keywords:** Bone infection, Fracture-related infection, *S. epidermidis*, *S. aureus*, Implant stability, Interleukin-17A

## Abstract

Biomechanical stability plays an important role in fracture healing, with unstable fixation being associated with healing disturbances. A lack of stability is also considered a risk factor for fracture-related infection (FRI), although confirmatory studies and an understanding of the underlying mechanisms are lacking. In the present study, we investigate whether biomechanical (in)stability can lead to altered immune responses in mice under sterile or experimentally inoculated conditions. In non-inoculated C57BL/6 mice, instability resulted in an early increase of inflammatory markers such as granulocyte-colony stimulating factor (G-CSF), keratinocyte chemoattractant (KC) and interleukin (IL)-6 within the bone. When inoculated with *Staphylococcus epidermidis*, instability resulted in a further significant increase in G-CSF, IL-6 and KC in bone tissue. *Staphylococcus aureus* infection led to rapid osteolysis and instability in all animals and was not further studied. Gene expression measurements also showed significant upregulation in CCL2 and G-CSF in these mice. IL-17A was found to be upregulated in all *S. epidermidis* infected mice, with higher systemic IL-17A cell responses in mice that cleared the infection, which was found to be produced by CD4+ and γδ+ T cells in the bone marrow. IL-17A knock-out (KO) mice displayed a trend of delayed clearance of infection (*P*=0.22, Fisher’s exact test) and an increase in interferon (IFN)-γ production. Biomechanical instability leads to a more pronounced local inflammatory response, which is exaggerated by bacterial infection. This study provides insights into long-held beliefs that biomechanics are crucial not only for fracture healing, but also for control of infection.

## Abstract (precis)

**Summary:** Physical movement between bone fragments after a fracture influence healing, and are shown here, for the first time, to influence immune responses and infection.

## INTRODUCTION

Fracture healing is a complex process influenced by several interconnected factors and processes, summarized in the so called ‘diamond concept’ (Giannoudis et al., 2007). Classically, the factors involved include osteogenic cells, an osteoconductive scaffold, growth factors and biomechanical stability. In recent years, the role of the immune system has emerged as an additional component of fracture healing (Claes et al., 2012; Schlundt et al., 2015; Godwin et al., 2017; Giannoudis et al., 2007; Ono and Takayanagi, 2017; Wendler et al., 2019). The first stage of the fracture healing cascade involves acute inflammation (Claes et al., 2012), and cells of the immune system such as T cells (Kalyan, 2016; El Khassawna et al., 2017; Schlundt et al., 2019; Bernhardsson et al., 2019), B cells (Sun et al., 2017a), macrophages (Ogle et al., 2016; Lu et al., 2017; Hozain and Cottrell, 2020) and granulocytes (Ramirez-GarciaLuna et al., 2017) are known to play a role throughout the process. In addition, their soluble mediators such as cytokines or chemokines (Edderkaoui, 2017; Sun et al., 2017b) are also known to play an important role in fracture healing.

Fracture-related infections (FRI) are a major complication in musculoskeletal trauma surgery, and are associated with increased hospital stays, long-term antibiotic treatment, multiple revision surgeries, and, overall, a reduced functional outcome (Metsemakers et al., 2018; Metsemakers et al., 2020). *Staphylococcus aureus* and the coagulase negative staphylococci (CoNS) are the leading etiologic agents in FRI (Kuehl et al., 2019). *Staphylococcus epidermidis* is the most prominent member of the CoNS in FRI and cause approximately 20% of all orthopedic device-related infections (Trampuz and Zimmerli, 2006). However, its prevalence may even increase to 50% in late-developing infections (Schafer et al., 2008), which is often attributed to the sub-clinical nature of *S. epidermidis* infections. Despite its prevalence in device-related infections, relatively little is known about host defenses against *S. epidermidis*. Innate immune responses to *S. epidermidis* have been studied outside the context of FRI and are known to involve the secretion of interleukin (IL)-6, IL-1β, tumor necrosis factor (TNF)-α or IL-8, most of which occurs upon toll-like receptor (TLR)-2 recognition (for review see Sabaté Brescó et al., 2017a). Currently, the specific details concerning adaptive immune responses to *S. epidermidis* are relatively unknown, although the use of immunodeficient mice has shown that mice lacking T and B cells have a delay in infection clearance (Vuong et al., 2004). IL-17A is a cytokine that, through the induction of chemokine production, attracts neutrophils to mediate defenses against different pathogens (Akdis et al., 2016). It has been shown to be involved in *S. aureus* infection clearance in mice (Chan et al., 2015; Henningsson et al., 2010; Yu et al., 2018), while it also seems to be important in human immune responses to *S. aureus* (Archer et al., 2016; Welch et al., 2015). Much less is known for *S. epidermidis* although some recent publications have shown that *S. epidermidis* exposure triggers IL-17A signaling pathways in human cells (McGuire et al., 2017). On the other hand, interferon (IFN)-γ, which has largely been explored in the context of *S. aureus* infections, has also been shown to be triggered by *S. epidermidis* in skin (Nakamizo et al., 2015).

Recently, we established a model of FRI in mice (Sabate Bresco et al., 2017b), in which we assessed the influence of biomechanical stability on infection progression. We could show that instability across a fracture leads to a delay in clearance of *S. epidermidis* from the tissues of C57BL/6 mice. The aim of the present study was to characterize the immune responses in mice with stable or unstable fixation and when inoculated with *S. epidermidis*, *S. aureus* or non-inoculated controls. We aimed to determine if the previously observed differences in infection due to biomechanical instability are linked to alterations in immune responses, as may be revealed by local secretion of soluble mediators, local and peripheral immune cell populations, or adaptive immune responses. The pro-inflammatory cytokine IL-17A was also investigated as a potential crucial factor for host response to FRI.

## RESULTS

### Cytokine/chemokine levels in non-inoculated versus infected bone

**Fig. 1.**
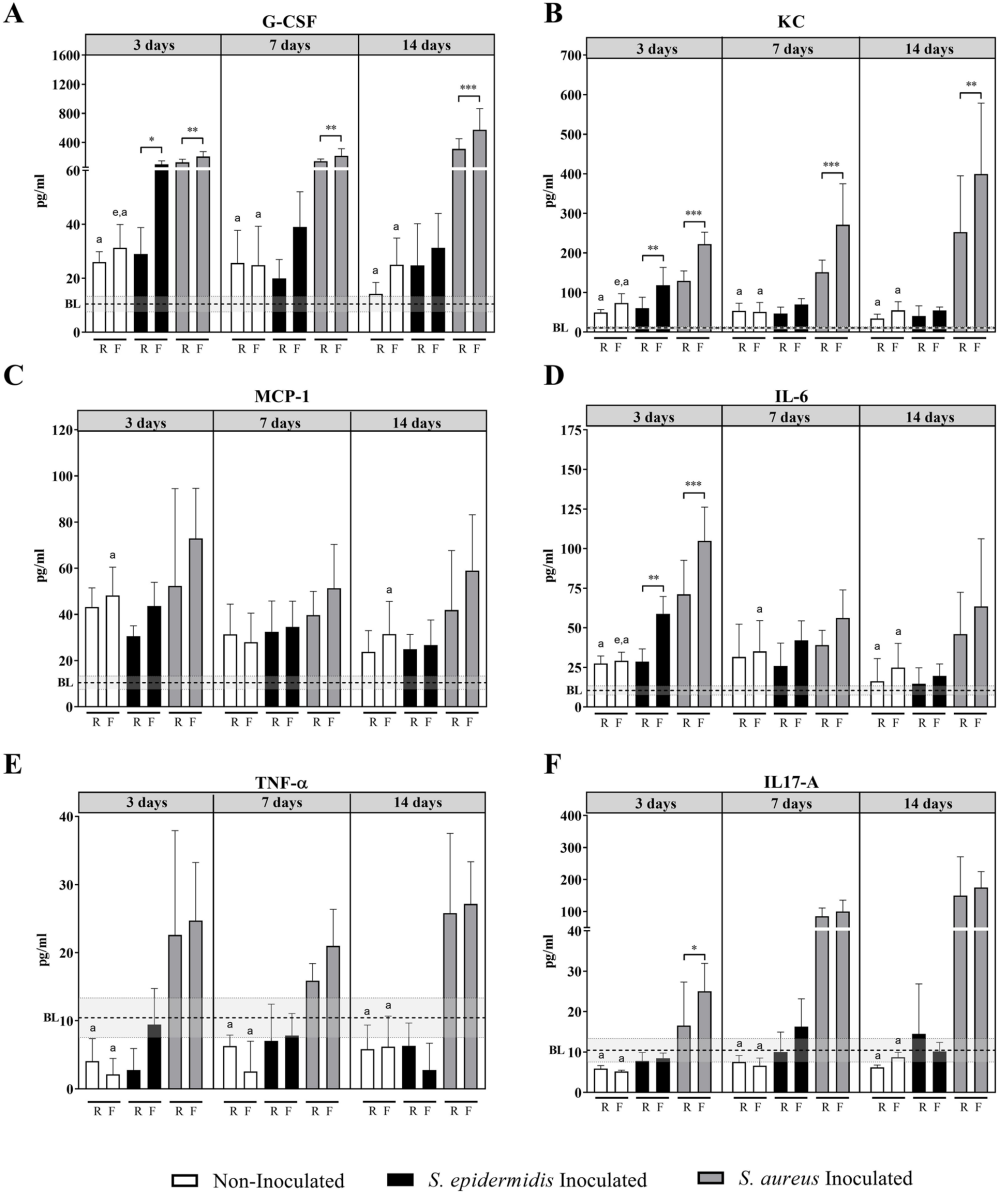
**Cytokine levels (pg/ml) in bone homogenate supernatants of C57BL/6 mice at 3, 7 and 14 days post-op.** (A) G-CSF, (B) KC, (C) MCP-1, (D) IL-6, (E) TNF-alpha and (F) IL-17A levels (pg/ml) in bone homogenates of non-inoculated, *S. epidermidis* inoculated and *S. aureus* inoculated mice, with rigif and flexible devices. Data shown are Mean+s.d. (*n*=5-11). BL: baseline, mean of the control group (non-operated mice); grey area: BL±s.d. of the control group. Two-way ANOVA per time point with Tukey’s post-hoc correction. Statistics summarize significant differences in the following comparisons: Non-inoculated versus *S. aureus* Inoculated (denoted by a); Non-inoculated versus *S. epidermidis* Inoculated (denoted by e); Rigid versus Flexible implant in each condition and time-point: *P>0.05, **P<0.01, ***P<0.001. R, rigid implant; F, flexible implant.

The fracture environment of non-inoculated C57BL/6 mice with rigid and flexible implants was characterized by analyzing several cytokines and chemokines in bone homogenate supernatants (Fig. 1). For non-inoculated C57BL/6 mice with both rigid and flexible fixation, innate immune response players granulocytecolony stimulating factor (G-CSF), keratinocyte chemoattractant (KC) monocyte chemoattractant protein (MCP)-1 and IL-6) increased above baseline at 3 and 7 days post-operatively (*P*<0.05) and returned to baseline levels between days 14 and 30 (Fig. 1A–D). For these non-inoculated mice, groups with a flexible device displayed a trend for higher levels of cytokines and chemokines; however, there were no statistically significant differences and levels were generally low.

Inoculation with *S. epidermidis* in C57BL/6 mice resulted in increased inflammation and innate immunity markers such as G-CSF and KC, as well as IL-6 (all *P*<0.05) with values peaking between days 3 and 7 followed again by a decrease closer to baseline levels between days 14 and 30 (Fig. 1). Inoculated animals with a flexible implant presented with significantly higher levels of cytokines and chemokines than those with a stable equivalent or non-inoculated counterparts, with statistically significant differences observed at day 3 for G-CSF, KC and IL-6 (*P*<0.05, Fig. 1A,B,D). TNF-α and IL-17A were often increased in *S. epidermidis* inoculated animals compared to non-inoculated ones, although differences were not statistically significant, while no differences were observed for IFN-γ (Fig. 1E).

Inoculation with *S. aureus* in C57BL/6 mice led to pronounced changes in most of the cytokines and chemokines studied, with G-CSF, KC, IL-6, TNF-α and IL-17A all being increased at all time-points studied (3, 7 and 14 days) (*P*<0.05, Fig. 1A,B,F). Once more, mice with a flexible implant displayed significantly higher G-CSF, KC and IL-6 levels when infected compared to animals with a rigid device (*P*<0.05). This was also observed for IL-17A at day 3. When *S. aureus* inoculated groups were compared to non-inoculated or *S. epidermidis* inoculated counterparts, significantly higher levels were observed for most of the markers.

Similar trends were observed in the BALB/c animals (Fig. S4). Again, when animals with a flexible implant were compared to their counterparts with a rigid implant, they presented higher levels of inflammation markers, such as IL-6, G-CSF or KC, reaching statistical significance for IL-6 and MCP-1 (*P*<0.05, Fig. S4).

Overall, the data presented above suggests that unstable conditions result in a higher inflammatory environment, which is exacerbated in the presence of an infection.

### Cell populations in the healing bone

**Fig. 2.**
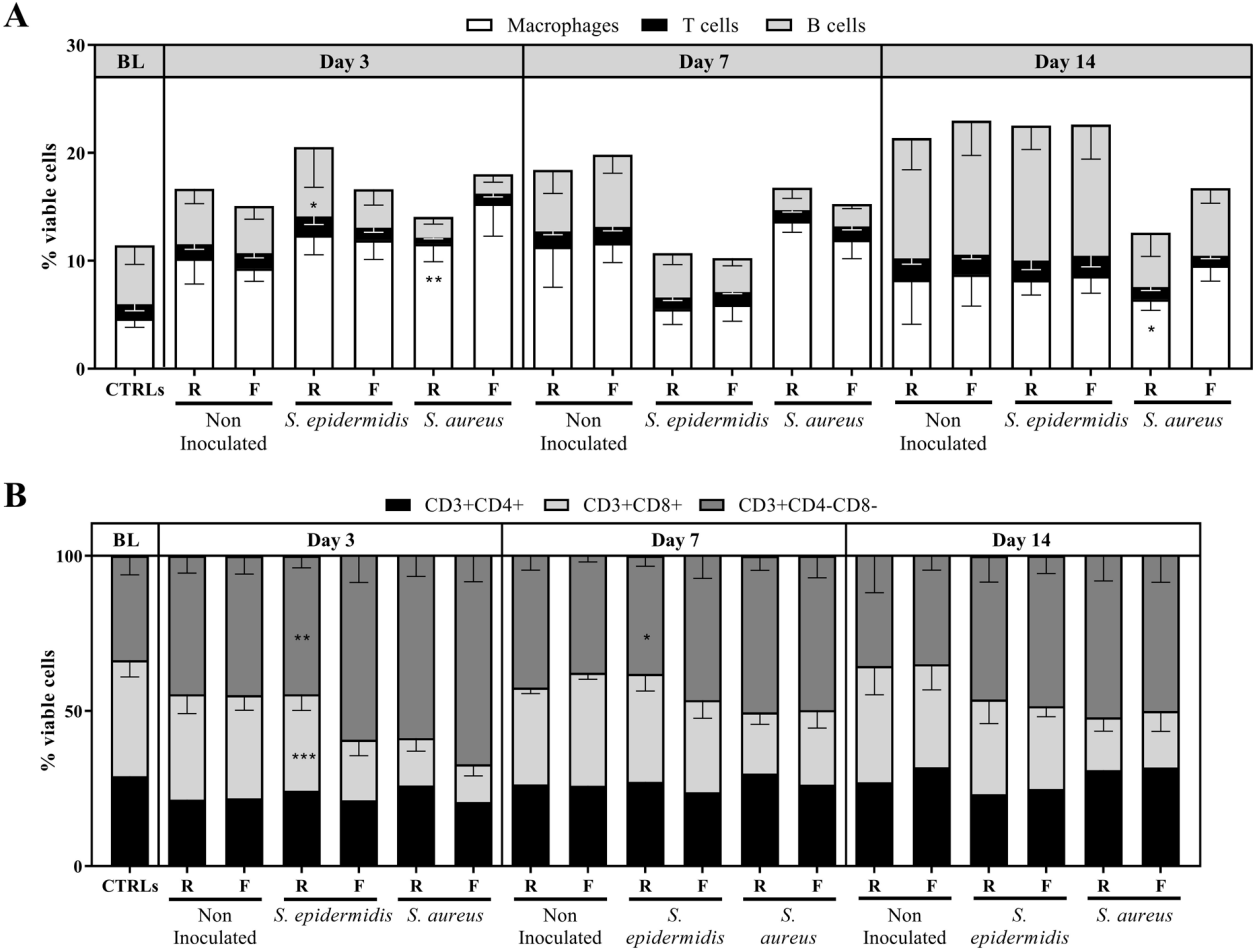
**Macrophage, T cell and B cell populations observed over time at the fracture site in C57BL/6 mice.** Upper panel, macrophage lineage cells (Ly6G-F4/80+), T cells (CD3+CD19-) and B lineage cells (CD19+CD3-) as a percentage of total viable cells (A). Lower panel, percentage of CD4+, CD8+ and CD4-CD8- calculated on CD3+ cell numbers (B), in bone single cell suspensions of C57BL/6 mice at days 3, 7 and 14 post-operatively. Mean values and SD (*n*=5-9). Two-way ANOVA per time point with Tukey’s post-hoc correction. Statistics summarize significant differences in the following comparisons: Non-inoculated versus *S. aureus* (denoted by a), Non-inoculated versus *S. epidermidis* denoted by e); Rigid versus Flexible implant within each condition: *P>0.05, **P<0.01, ***P<0.001. BL: baseline, mean of the control group (non-operated). R, rigid implant; F, flexible implant.

Immune cell populations in the single cell suspension from bone homogenates were analyzed by flow cytometry (Fig. 2). Cells of the macrophage lineage (F4/80+Ly6G−), T cell lineage (CD3+CD19−) and B cell lineage (CD19+CD3−), as well as T lymphocyte subclasses (CD4+, CD8+ and CD4-CD8−), were analyzed at days 3, 7 and 14. As no significant differences were observed in total cell counts and viability, results are reported as a proportion of total live cells or as a percentage of total CD3+ cells, as appropriate. Comparisons focused on the rigid versus flexible fixation in each condition, as well as on the effect of bacteria inoculation.

Macrophage lineage cells, T lineage cells and B lineage cells are shown in Fig. 2A. In operated but non-inoculated C57BL/6 mice, macrophage proportions were seen to increase at early time points post-surgery (day 3 and 7) and they returned to baseline levels after day 14, while T and B lineage cell proportions peaked at day 14. No significant differences were observed between rigid and flexible plates. For *S. epidermidis* inoculated C57BL/6 mice, an increase in macrophage lineage cells was also observed at day 3, with a significant reduction thereafter (*P*<0.05), and B cells again peaked at day 14. Few significant differences were observed between rigid and flexible plates. Finally, for *S. aureus* inoculated animals, similar observations were noted for macrophage lineage cells, with an increase observed at day 3, which was significant compared to non-inoculated counterparts (*P*<0.05). At day 3 and at day 14, significant differences were observed when comparing rigid and flexible plates, with a significant difference at day 14 (*P*<0.05). B lineage cells in *S. aureus* inoculated mice showed an opposite trend to both non-inoculated and *S. epidermidis* inoculated mice, with a significant decrease in the proportions observed at all time-points (*P*<0.05). T lineage cells were also often decreased in *S. aureus* inoculated compared to non-inoculated.

Within the T cell subclasses (shown in Fig. 2B), an increase in CD4-CD8- T cells was observed at day 3 for non-inoculated C57BL/6 mice, but proportions subsequently returned to baseline levels and (in)stability did not impact any of these observations. *S. epidermidis* inoculated animals with a flexible device experienced a significant increase in CD4−CD8− T cells compared to *S. epidermidis* inoculated mice with rigid plate at early time-points, in line with a decrease of CD4−CD8+ cells (*P*<0.05)*. S. aureus* inoculated animals also experienced an increase of CD4−CD8− T cells, regardless of implant type (*P*<0.05, Fig. 2B). Differences with non-inoculated equivalents were maintained at all time-points.

Similar trends were observed in BALB/c animals, with recruitment of macrophages at early time points, and a peak in T and B cells observed at day 14 (Fig. S6). Within T cell populations, there was an increase of CD4-CD8- in *S. epidermidis* inoculated mice, similar to that observed in C57BL/6 mice (*P*<0.05). Relatively few differences were observed when comparing rigid and flexible plates. Finally, as a difference between mice strains, we observed that the balance between CD4 and CD8T cells was more skewed towards CD4 in BALB/c mice compared to C57BL/6 mice.

Additionally, the local environment was also characterized at day 3 with myeloperoxidase (MPO) measurements in bone homogenates of C57BL/6 mice, as an indirect way to assess early granulocyte infiltration (Fig. S5). Significantly higher levels of MPO relative to baseline were detected for flexible implant groups, although this did not reach significance relative to rigid equivalents (*P*<0.05).

### Gene expression in C57BL/6 mice

**Fig. 3.**
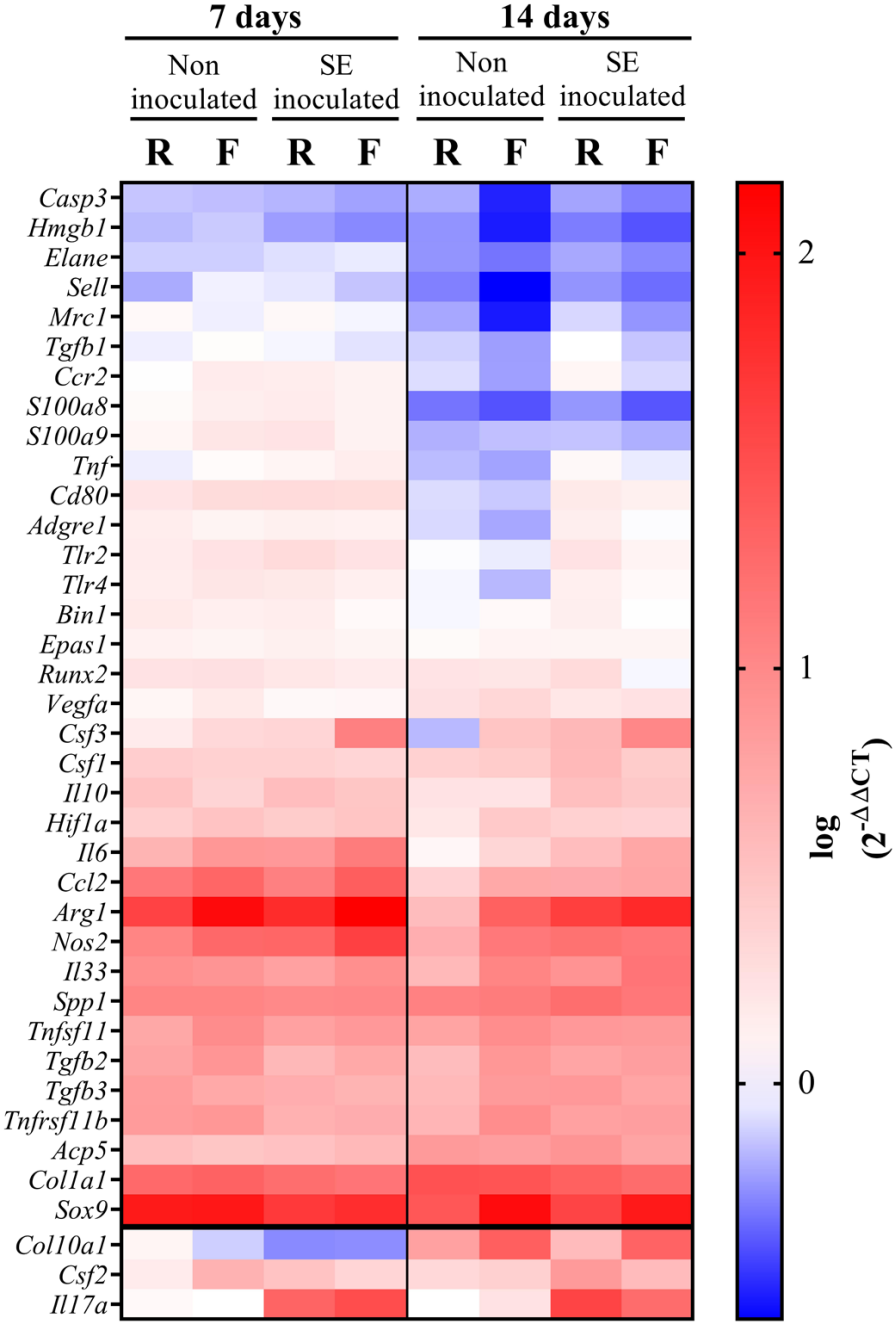
**Heat map of gene expression at days 7 and 14 in inoculated and *S. epidermidis* inoculated C57BL/6 mice with rigid and flexible fixation.** Values represented are the average log (2^−ΔΔCT^) of each group (*n*=5–6). Control group (non-operated) was used as a calibrator except for *Col10a1*, *Csf2* and *Il17a* (bottom of the chart) where Non-inoculated Rigid group was used, since no gene expression was detected in the Control group. SE, *S. epidermidis*.

mRNA expression was analyzed at days 7 and 14 in non-inoculated and *S. epidermidis*-inoculated C57BL/6 mice with rigid and flexible implants. Results are summarized in Fig. 3, with detailed charts for selected genes of interest, and statistical evaluation, included as Figs S7 and S8. Expression was detected for most of the genes; however, *Col10a1*, *Csf3* and *Il17a* were only expressed in operated femurs (not in control mice).

From the mRNA expression data, several patterns could be observed (Fig. 3). Firstly, a group of genes showed a consistent increase over time in all operated animals (non-inoculated and *S. epidermidis* inoculated) with highest levels detected at day 14 (Fig. 3; Fig. S7). These were often linked to bone formation and included *Col1a1*, *Acp5*, *Col10a1*, *Runx2*, *Sox9* and *Spp1*. Both *Col10a1* and *Sox9* were significantly further upregulated in animals with a flexible device compared to mice with a rigid device. A second group of genes, associated with the on-going inflammatory process, were maximally expressed at day 7 in all groups and remained upregulated at day 14. This included genes such as *Arg1*, *Ccl2*, *Nos2*, *Il6* and *Hif1a* (Fig. 3; Fig. S8). Of note, these often were upregulated in flexible implant groups compared to rigid counterparts. A third group of genes, associated with immune responses and pathogen recognition, decreased over time in non-inoculated animals, independently of implant type, while they remained slightly upregulated in inoculated mice. Such genes included *Ccr2*, *Cd80*, *Il10*, *Tlr2*, *Tlr4*, *Tnf* and *Adgre1*. No significant differences were observed between rigid and flexible implants except for *Adgre1* at day 14, which was higher in the inoculated flexible plate group versus its rigid counterpart. *Il17a* was almost exclusively expressed in inoculated animals, with no detection or very low levels in non-inoculated mice. A final group of genes were downregulated at all time points in all operated femurs (e.g. *Casp3*, *Elane*, *Hmgb1* and *Sell*), or were downregulated at day 14 (*Mrc1*, *S100a8*, *S100a9* and *Tgfb1*). Interestingly several of these genes are associated with cell damage signaling or neutrophil products.

In terms of bacterial burden in these mice, animals with a rigid device tended to clear the infection more quickly than animals with a flexible implant (Fig. S9), which was consistent with earlier findings (Sabate Bresco et al., 2017b).

### Systemic immune responses associated with infection

**Fig. 4.**
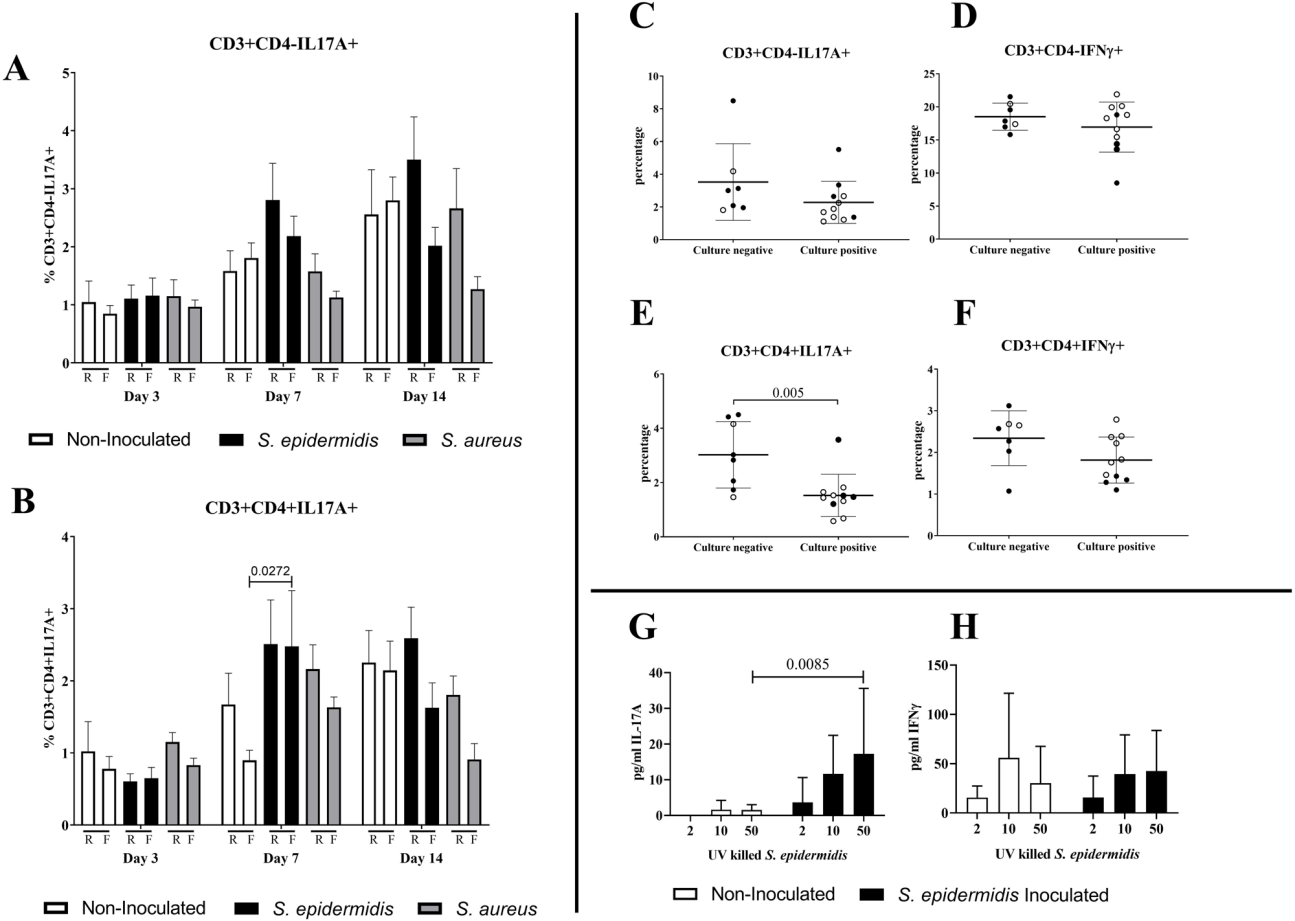
**Systemic immune responses associated with infection.** (A,B) Percentages of CD3+CD4-IL-17A+ and CD3+CD4+IL-17A+ T cells in popliteal lymph node single cell suspensions, for both rigid (R) and flexible (F) plate groups. Data shown are Mean+s.d. (*n*=6–9). Two-way ANOVA with Tukey’s post-hoc correction per time-point. (C–F) Percentage of IL-17A+ and IFN-γ+ T lymphocytes in popliteal lymph nodes at day 14 in culture-negative mice (infection cleared) or culture-positive mice (infected). Both rigid and flexible samples grouped together (*n*=7–11), (black circles for rigid and empty circles for flexible). Mann–Whitney test. (G) IL-17A and H) IFN-γ production by splenocytes from non-inoculated and *S. epidermidis* inoculated C57BL/6 mice at day 30 (rigid and flexible samples grouped together) after stimulation with UV-killed *S. epidermidis in vitro* (dose indicated in the x-axis: ratio of bacteria per spleen cell). Data shown are Mean+s.d. (*n*=5–8). Two-way ANOVA with Sidak post-hoc correction.

To evaluate adaptive immune responses, popliteal lymph node single cell suspensions were stimulated for 4 h with phorbol 12-myristate 13-acetate (PMA), ionomycin and brefeldin A, and stained for surface markers and intra-cellular cytokines. In all groups, an increase in the proportion of cytokine-producing T cells was observed after day 3 (*P*<0.05, Fig. 4A,B for IL-17A; data not shown for IL-10 and IL-4). In terms of differences related to implant type, CD3+CD4+IL-17A+ T cells were significantly increased when *S. epidermidis* inoculated mice with a flexible implant were compared to their non-inoculated counterpart (*P*<0.05). Otherwise, no significant differences were observed (Fig. 4A,B).

Adaptive responses of *S. epidermidis*-inoculated mice at day 14 were compared between animals that had cleared infection (culture negative for all plated samples) versus culture positive animals (Fig. 4C–F). Culture-negative mice displayed a significant increase in proportions of IL-17A producing CD4+ T cells (*P*<0.005), and a similar trend was observed for IFN-γ producing CD4+ T cells, although this did not reach statistical significance (*P*=0.1259).

In order to address specific responses to *S. epidermidis*, we stimulated splenocytes with UV-inactivated *S. epidermidis*, for both non-inoculated and *S. epidermidis* inoculated mice at day 30, in C57BL/6 (Fig. 4G,H) and in BALB/c mice (Fig. S10). After 72 h, secretion of several innate immunity markers was observed for both non-inoculated and inoculated groups. IL-17A was the cytokine most differentially expressed between both groups (non-inoculated versus *S. epidermidis* inoculated), being almost exclusively secreted by animals that had been infected with *S. epidermidis* (Fig. 4G) for both C57BL/6 and BALB/c strains. In addition, IFN-γ was also significantly increased in *S. epidermidis* inoculated BALB/c mice (*P*<0.05, Fig. S10E,F).

### Role of IL-17A in infection progression

As IL-17A seemed to be selectively induced by the presence of *S. epidermidis* and IL-17A+ T cells were increased in lymph nodes of mice that cleared infection (Fig. 4E), IL-17A knock-out (KO) mice were used to study the role of IL-17A in infection clearance. Histological analysis was performed to assess bone healing and infection; however, we did not observe any major differences between wild-type (WT) and IL-17A KO animals with that approach (Fig. S11).

**Fig. 5.**
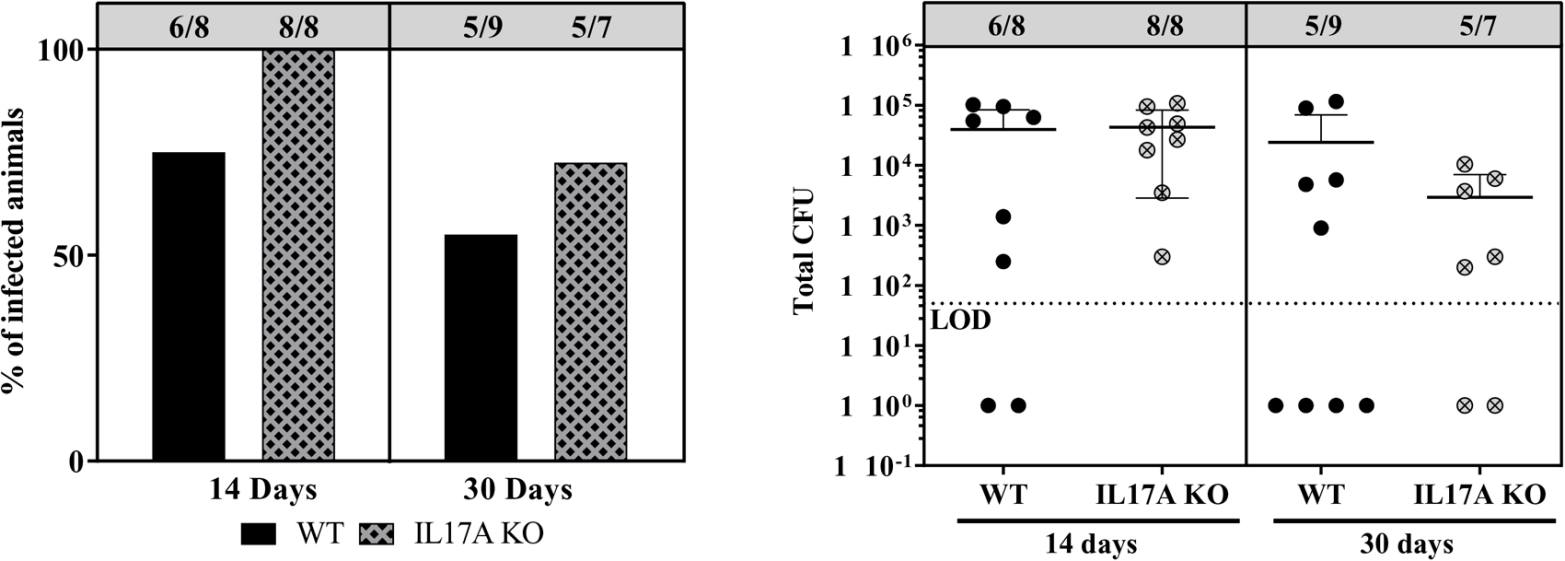
**Bacteriological outcome of IL-17A K/O mice versus WT controls.** Percentage of WT and IL-17A KO C57BL/6 mice infected at days 14 and 30 (left panel), and total Colony Forming Units (CFU) counts (sum of CFU from bone, soft tissue and implant; right panel). Data shown are Mean+s.d. (*n*=7–9). LOD: Limit of detection (0.5×10^2^) culture negative samples are represented as 1. Categorical infected versus not infected was assessed by Fisher's exact test, and quantitative CFU data was compared by Mann–Whitney test.

Thus, we proceeded to perform a study (Table S3) in order to assess infection outcomes and related immune responses in the absence of IL-17A. Once again, some WT animals (25%) had cleared the infection by day 14, although none of the IL-17A KO animals cleared the infection at that time point (Fig. 5). However, differences were not statistically significant (*P*=0.22, Fisher’s exact test). At day 30, some of the WT mice were again less often infected, however, IL-17A KO animals had also begun to clear the infection at this time. No significant differences were observed at any time point in terms of bacterial counts.

**Fig. 6.**
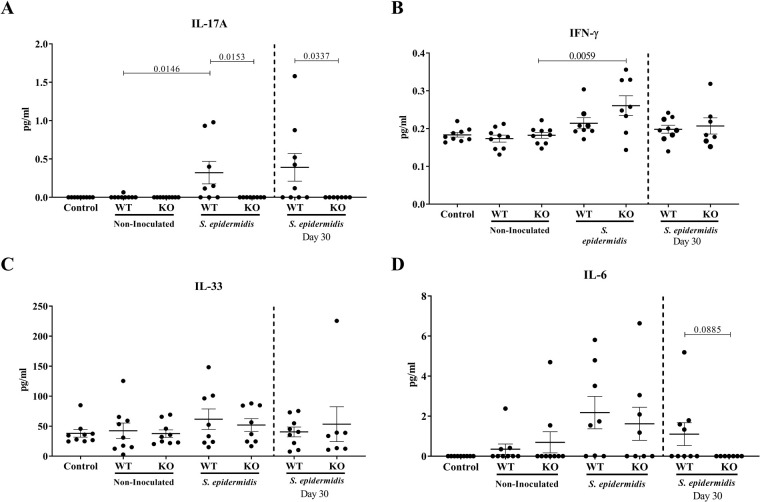
**Cytokine levels in bone homogenates of control, WT and IL-17A KO C57BL/6 mice.** (A) IL-17A, (B) IFN-gamma, (C) IL-33 and (D) IL-6 levels (pg/ml) in bone homogenates of control, WT and IL-17A KO, non-inoculated and *S. epidermidis* inoculated. Data shown are Mean±s.d. (*n*=7–9). Two-way-ANOVA with Tukey’s post-hoc for day 14, Mann–Whitney test for day 30.

The healing bone environment was again assessed by measuring cytokines and chemokines in WT and KO mice (Fig. 6). In WT mice, IL-17A was significantly increased when compared to non-inoculated animals (Fig. 6A) and, as expected, was not present in KO mice.

There was a trend for IFN-γ, IL-33 and IL-6 to increase in *S. epidermidis* inoculated mice but this was not significant. In KO mice, except for IL-17A, which was not present, similar findings were observed, with a trend for increased IL-6 and a significant increase of IFN-γ, possibly as a compensatory response to the lack of IL-17A. At day 30, cytokine levels were similar between WT and KO with the exception of IL-17A. Once more, IL-17A levels tended to be reduced in mice that had cleared the bacteria.

T cell populations in popliteal lymph node were also studied. No significant differences were observed for IFN-γ, IL-10 or IL-4 producing T cells between WT and KO animals (data at day 14 shown in Fig. S12).

### Source of IL-17A in bone marrow

**Fig. 7.**
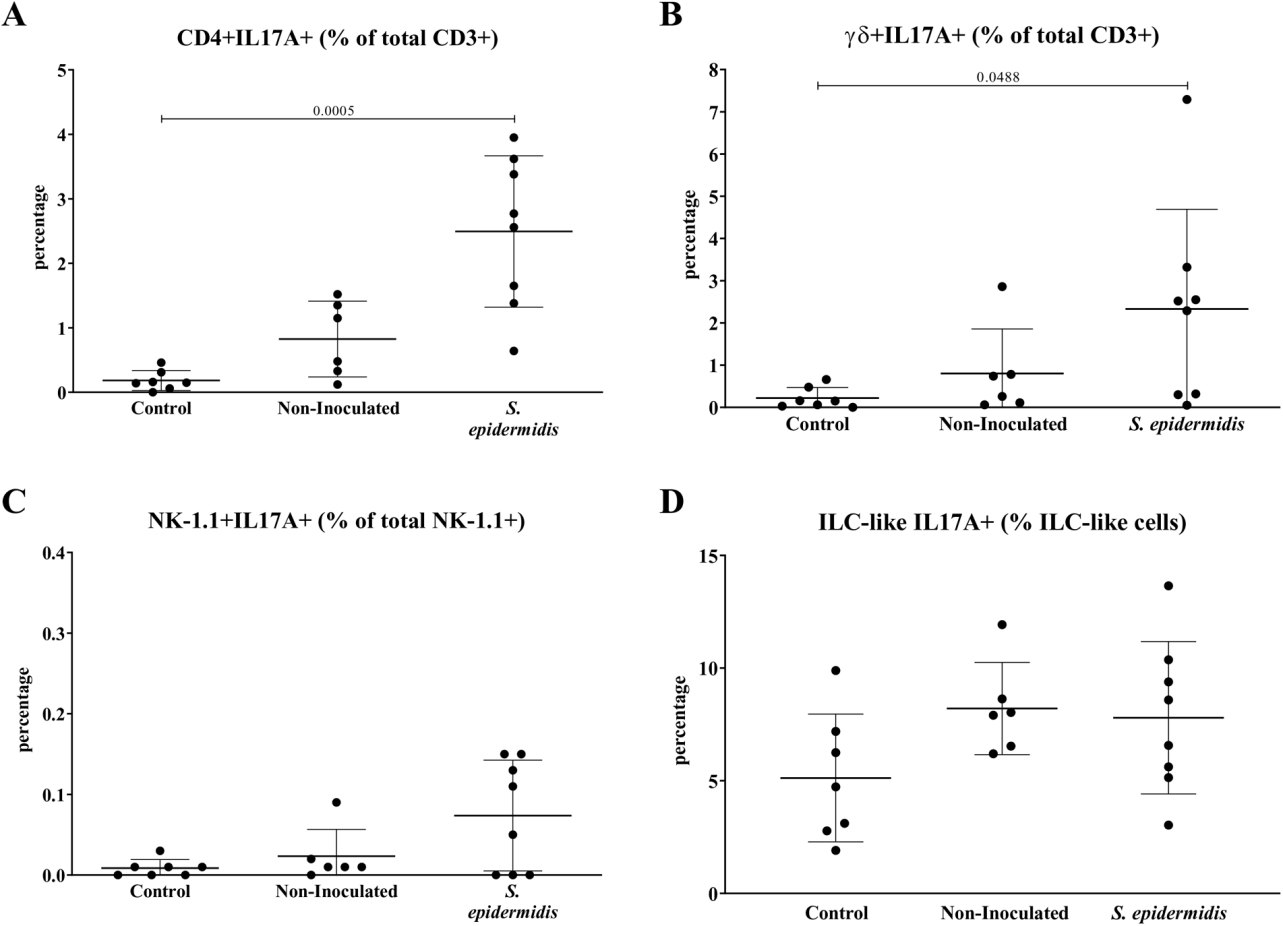
**Percentage of IL-17A producing cells in control, non-inoculated with a rigid implant or *S. epidermidis* inoculated with a rigid implant for WT mice at day 14.** Percentage of (A) CD3+CD4+IL-17A+ T cells, (B) CD3+TCRγδ+IL-17A+ T cells, (C) CD3-NK-1.1+IL-17A+ cells and (D) ILC-like cells in control mice, non-inoculated with a rigid implant mice and *S. epidermidis* inoculated with a rigid implant mice. Each graph title shows over which population percentage is expressed. Data shown are Mean±s.d. (*n*=7-11). One-way-ANOVA or Kruskal–Wallis, with Tukey’s or Dunn's post-hoc test.

We then aimed to identify the cellular source of the IL-17A detected in bone. We observed several cell types in bone homogenates of C57BL/6 WT that produced IL-17A such as CD4+ T lymphocytes, γδ T lymphocytes, NK cells and lineage-negative cells (Fig. 7). Following surgery, there was a trend for IL-17A+ CD4+ and γδ T lymphocytes to increase in both non-inoculated and *S. epidermidis* inoculated mice compared to control groups, being significant in mice inoculated with *S. epidermidis*.

## DISCUSSION

In this study we aimed to characterize the immune responses associated with healing bone in different biomechanical contexts and in the presence or absence of *S. epidermidis* and *S. aureus* FRI. Our model included implants with different mechanical properties, which, based on our previous findings (Sabate Bresco et al., 2017b), can lead to different kinetics of bone healing, and, crucially, infection clearance. Our past data was the first to show that, under controlled conditions, *S. epidermidis* inoculated C57BL/6 mice were more likely to clear infection when a rigid fixation device was implanted compared to a flexible device: results that were confirmed in this study. With this study, we sought to investigate if differential immunological responses to fixation stability could explain the interaction of biomechanical stability and infection clearance.

The observation that non-inoculated animals with a flexible implant tended to secrete higher levels of inflammatory markers (e.g. IL-6, G-CSF and KC), compared to mice with a rigid implant (both in C57BL/6 and BALB/c), suggests that instability leads to a more inflamed local microenvironment. This was also supported by MPO analysis in bone marrow and in gene expression analysis. Similar findings have been reported in a sheep model of impaired bone healing (Schmidt-Bleek et al., 2012). In that study, two types of fixation were also used, a more stable and a less stable one that impaired bone healing. A higher percentage of cytotoxic T cells and macrophage lineage cells were observed in the hematoma and bone marrow of less stable fractures, which were suggested to be associated with a higher/prolonged inflammatory environment (Schmidt-Bleek et al., 2012). Thus, in non-inoculated conditions, the more inflamed environment observed in the unstable context could have a negative impact on bone healing. Consistent with this, the link between increased inflammation (acute or chronic) and fracture healing complications has already been proposed in pre-clinical studies and in human patients (Alblowi et al., 2009; Abou-Khalil et al., 2014; Reikeras et al., 2005; Recknagel et al., 2011; Claes et al., 2017; Hurtgen et al., 2016). In a clinical setting, polytrauma patients, who often have high systemic levels of inflammatory cytokines (such as IL-6 or TNF-α), present with a high percentage of fracture healing complications (Hildebrand et al., 2016). Also, pronounced inflammatory responses were observed in hematomas (at 72 h) of patients considered immunologically compromised (e.g. patients with autoimmune diseases, cancer or osteoporosis), which often exhibit delayed healing and other complications (Hoff et al., 2017). While inflammation is required to initiate the healing process, the timely resolution of this inflammation is believed to be necessary for effective healing to occur (Claes et al., 2012). Therefore, the prolonged inflammatory microenvironment due to the use of a flexible plate (unstable context) can be a factor contributing to a higher risk of delayed or non-union often associated with instability.

In our model, we have also characterized the local fracture environment and the role of stability for affecting the host response to infection. For *S. epidermidis*, differences at the cytokine or gene expression level between non-inoculated and inoculated animals were often not statistically significant. This data supports the concept that our model closely resembles a low-grade infection, also described in other models (Lovati et al., 2016; Lovati et al., 2017) and in human patients (Metsemakers et al., 2016; Seebach and Kubatzky, 2019). The only exception was mice with flexible implants at early time points (both for C57BL/6 and BALB/c), which showed significantly higher levels of inflammatory markers such as IL-6, G-CSF or KC. Although we did not specifically identify the cellular sources of these cytokines/chemokines, macrophages present in the tissue, and also osteoblasts, have been shown to secrete similar cytokines upon *S. epidermidis* stimulation (Strunk et al., 2012; Wilsson et al., 2008; Dapunt et al., 2016). At later time-points, cytokine levels in all operated mice decreased to levels observed in control mice, although inoculated mice that remained culture positive often continued to secrete higher cytokine levels. The gene expression data in non-inoculated and *S. epidermidis* inoculated C57BL/6 mice are consistent with the cytokine quantification data described above. We again observed a trend for higher inflammatory/tissue damage markers (e.g. *Il6*, *Ccl2* or *Il33*) in animals with a flexible implant compared to rigid counterparts. Furthermore, the combination of flexible implant and *S. epidermidis* infection led to the highest responses regarding cytokine/chemokine release (e.g. *Csf3*, *Il6*, *Nos2* or *Ccl2*). Interestingly, the increased expression of *Hif1a* suggests that the instability and/or infection results in a more hypoxic environment, although this did not translate to significant differences in hypoxia-inducible factor (Hif)-1α regulated genes such as *Vegfa*, which could suggest an inadequate adaptation to hypoxia as suggested by Hoff et al. (Hoff et al., 2011). Similar findings were observed for *Vegfa* and *Vegfc* in a murine model comparing the same type of implants (Montjovent et al., 2013). Further studies to investigate the responses of other Hif-1α regulated genes, such as *Ldha* or *Pgk1*, are therefore warranted to elucidate the role of hypoxia in unstable fixation associated with FRI. Taken together, we observed that the combination of a flexible implant and infection results in higher levels of inflammation, hypoxia and tissue damage markers. These observations, reported for the first time in the context of infection and instability, could potentially impair immune responses and be detrimental for infection clearance. This concept matches a recent summary of the historical and clinical thinking in relation to stability and infection, whereby a vicious cycle of instability and infection are often interdependent (Foster et al., 2021). The present study provides some new insights and mechanisms underlying this long-held clinical interpretation.

A number of important differences in the immune response were observed following inoculation with *S. aureus* compared to *S. epidermidis*. *S. aureus* inoculation led to a much higher and sustained inflammation in the tissue, in line with the acute nature of *S. aureus* infections and the consequent host immune response to the infection (Sabate Bresco et al., 2017b). For example, almost all cytokines measured (IL-6, G-CSF, KC, MCP-1) were significantly increased in *S. aureus* inoculated mice, up to day 14, when compared to non-inoculated or *S. epidermidis* inoculated mice. This highly inflamed environment was accompanied by significant bone destruction. Similar to other studies, we observed high levels of IL-17A in the infected tissues (Rochford et al., 2016; Prabhakara et al., 2011a), a hallmark of TH17 responses and a cytokine gaining relevance in *S. aureus* immune responses (Blanchette et al., 2017). IL-17A may be relevant for infection clearance, but has also been suggested to contribute to pathogenesis due to the stimulatory effects of IL-17A on bone destruction and tissue damage (Prabhakara et al., 2011b; Jensen et al., 2015).

As a summary of the findings described above, cytokine secretion in full bone homogenates early after surgery depicted an increase for several innate immune markers (such as G-CSF, TNF-α or IL-6) in all groups, often increased in animals with a flexible implant compared to animals with a rigid implant. This was especially evident upon infection with *S. epidermidis* or *S. aureus*. It can be hypothesized that an excessive or prolonged inflammatory environment may contribute to the delayed bacterial clearance, as others also suggested (Seebach and Kubatzky, 2019), observed in unstable fractures. However, the specific contribution of inflammation *per se* to delayed bacterial clearance is difficult to assess, since inflammatory responses are directly induced by the trauma of the surgical procedure and the infection itself. In future, studies involving anti-inflammatory treatments may help to elucidate inflammation requirements for successful clearance of bacteria, although this needs to be carefully addressed as fracture repair cascade and recruitment of immune cells to the site for infection resolution require such signals to be initiated. In addition, it would also be interesting to combine the fracture-model presented here with other disease models (such as obesity or diabetes) together with infection in order to determine how fixation stability influences infection progression in groups of patients known to be at higher risk of post-surgical complications.

At the cellular level, we observed an increase of macrophage lineage cells in the bone marrow/fracture site at early time-points, in line with the peak of innate immune markers. Despite the fact that we could not detect mature neutrophils directly, MPO measurements also suggested recruitment of neutrophils at day 3. Conversely, the recruitment/accumulation of adaptive immune cells (T and B cells) peaked later, at day 14. This is consistent with previous observations, where two sequential recruitment waves of T and B cells were observed during bone healing (Konnecke et al., 2014). Regarding immune cell subclasses, an increase of double negative (CD4-CD8-) cells was noted at day 3, especially in the flexible plate, *S. epidermidis* inoculated group, and *S. aureus* inoculated animals (both rigid and flexible implants). The fact that these three groups demonstrated higher inflammatory responses suggests that different levels of inflammatory markers may alter the pattern of cells recruited. Of note, γδ T cells, which are CD3+ and frequently CD4-CD8- (Hayday, 2000), have been associated with *S. aureus* infection (specifically peritonitis and surgical site infection models) responses, at least at early time-points (Murphy et al., 2014; Maher et al., 2013). Currently it is unknown whether γδ T cells play any role in *S. epidermidis* infection although it has been shown that they are induced at least in skin upon pre-treatment (Strbo et al., 2020), so further studies regarding their role and an understanding of relevant subtypes are required.

We observed that within the leukocyte compartment in bone marrow at the fracture site, T helper cells and γδ T cells were the main sources of IL-17A. IL-17A producing lymphocytes have been proposed to contribute to bone healing (Ono et al., 2016; Croes et al., 2016; Kokubu et al., 2008) but also to bone destruction (Uluckan et al., 2016; Kim et al., 2014). Interestingly, in our study, these cells were also detected in non-inoculated animals suggesting that in fact they may play a role in bone healing. However, in those mice, cell number and *Il17a* gene expression were very low, which could be linked to time points studied; as in other studies IL-17A has been shown to peak 24–48 h after fracture and their levels are reduced over time (Ono et al., 2016). Nevertheless, we performed histology in IL-17A KO mice and we did not observe major differences in terms of bone healing when compared to WT mice at days 14 and 30 post fracture (Fig. S11; and Sabate Bresco et al., 2017b). Upon infection, it was observed that IL-17A was induced at both the mRNA and the protein level, as well as in adaptive immune cells, suggesting a potential role for TH17 responses during infection. On the other hand, local levels of IL-17A in culture negative mice were lower than the average, which could be linked to the resolution of the infection or could indicate a role in active infection. When addressing role of IL-17A in Staphylococcal infections, similar observations (induction upon infection) have been previously reported in *S. aureus* FRI (Rochford et al., 2016) and there is evidence that TH17 responses are important for *S. aureus* clearance in other infection models (Montgomery et al., 2014; Vidlak and Kielian, 2012; Maher et al., 2013; Yu et al., 2018), or in reducing *S. aureus* nasal carriage (Archer et al., 2016). Nevertheless, other studies have suggested a more detrimental role of this cytokine in *S. aureus* FRI (Prabhakara et al., 2011b). Concerning a role for TH17 cells in *S. epidermidis* infection, there is currently limited data to support such a role, although it has been shown in a foreign-body related infection that adaptive immunity is required for infection clearance (Vuong et al., 2008). The potential role of IL-17A-associated responses in our model was investigated using IL-17A KO mice. Since IL-17A is involved in neutrophil recruitment and activation, this may indicate a beneficial effect of IL-17A in clearing bacterial infection, although the pro-inflammatory and pro-osteolytic effects of IL-17A in murine models of inflammatory arthritis is suggestive of additional tissue damaging effects (Roark et al., 2007). This has also been proposed for *S. aureus* infections, suggesting that bacteria skew adaptive immunity towards a TH17/TH1 profile in order to evade the immune system (Jensen et al., 2015). We observed that there was a trend for IL-17A KO animals to clear the *S. epidermidis* infection less effectively than in WT animals, although differences were not statistically significant. While IFN-γ was not significantly increased in WT animals following infection, we did observe an increase in the secretion of this cytokine in IL-17A KO animals, suggesting a compensatory mechanism requiring more robust IFN-γ responses in the absence of IL-17A. IL-17F, which has a high homology with IL-17A and can signal through the same receptor (Akdis et al., 2016) or IL-22, also produced by TH17 cells, would be other candidates with the potential to compensate for IL-17A loss of function; however, we did not find detectable levels of IL-17F or IL-22 cytokines in WT or KO mice. Moreover, other studies have demonstrated a positive role for IFN-γ in *S. epidermidis* infections (Boelens et al., 2000). This would suggest a need for both TH1 and TH17 responses, as reported in some studies with *S. aureus* (Lin et al., 2009; Ferraro et al., 2019), which should be assessed in future studies. Nevertheless, due to the osteolytic effect of cytokines such as IL-17A, it may be expected that immunotherapeutic strategies aimed at modulating IL-17 should be carefully considered in the context of infectious disease. Furthermore, since we only used the rigid plate in the IL-17A KO animals; it is currently unknown whether we would observe enhanced pro-inflammatory responses using the flexible implant, which may influence the relative efficacy of IL-17A.

Finally, we had previously observed that C57BL/6 mice cleared infection earlier than BALB/c mice. Although it was not the main aim of this study and thus an in-depth analysis was not performed, we did observe some differences in terms of immune responses between C57BL/6 and BALB/c strains. We observed that BALB/c lymphocyte populations were more skewed towards CD4+ T cells in lymph nodes, with lower percentages of IFN-γ and IL-17A producing T helper cells when compared to C57BL/6 mice. Similar observations were seen in bone marrow, where BALB/c mice presented higher percentages of CD4+ T cells while CD8+ T cells were more frequent in C57BL/6 mice. Further studies would be required to elucidate the exact mechanisms leading to the differences observed in terms of infection clearance, but nevertheless, host background is an important consideration when performing pre-clinical studies.

### Conclusion

Our data suggests that fracture instability leads to an increase in inflammatory cytokines locally, which was supported by findings in both C57BL/6 and BALB/c genetic backgrounds. The combination of *S. epidermidis* infection and instability led to a significant increase in several inflammatory markers at early time-points, with a trend being maintained at later time-points. *S. aureus* infections resulted in a much more severe inflammatory response, associated with the higher secretion of pro-inflammatory cytokines. A trend for higher inflammation during *S. aureus* infection was also observed in animals with a flexible implant. However, since all animals remained infected in the *S. aureus* group, it is currently not possible to hypothesize how mechanical stability influences *S. aureus* clearance. Levels of inflammation were associated with different patterns of local immune cell recruitment, but no significant differences were observed systemically. Finally, the role of IL-17A in infection clearance was addressed, with the use of IL-17A KO animals. Bacterial clearance was modestly impaired in the IL-17A KO animals but differences to WT animals were not statistically significant, perhaps due to compensatory increases in IFN-γ/TH1 responses.

## MATERIALS AND METHODS

### Animals

The study was approved by the ethical committee of the canton of Graubünden in Switzerland (TVB 11/2013 and TVB 2016/03) and was carried out in a research facility accredited by the Association for Assessment and Accreditation for Laboratory Animal Care (AAALAC) International. Skeletally mature (20–28 weeks old, average weight±s.d.: 24.69±2.32 g), female, specific pathogen free (SPF) C57BL/6 and BALB/c mice, purchased from Charles River (Germany), and C57BL/6 IL-17A KO, kindly provided by Prof. Dr. Manfred Kopf (ETH Zürich, Switzerland), were used in this study. Younger mice were not considered due to the need for skeletal maturity in this fracture healing study, and males were not considered due to increased risk of implant failure due to heavier bodyweight. All animals were acclimatized to experimental conditions for at least two weeks prior to the start of the study. Mice were housed under a 12-h dark/ 12-h light cycle, in groups of three to six in individually ventilated cages (XJ, Allentown) that were changed weekly. Mice were re-housed in the same groups post-surgery. Animals were fed with a standard diet (3436, Provimi Kliba, Switzerland) and had free access to water.

### Study design

A full description of study groups, time points and group size is available in Tables S1–S3. In brief, the first series of experiments consisted of the assessment of immune responses in C57BL/6 and BALB/c mice receiving rigid and flexible implants (non-inoculated, *S. epidermidis* and *S. aureus*-inoculated) (Sabate Bresco et al., 2017b). The follow-up experiments focused only on C57BL/6 mice with *S. epidermidis* infection due to the extended osteolysis when *S. aureus* infection was present (thereby eliminating stability differences between groups). In these mice, mRNA expression was measured in non-inoculated and *S. epidermidis*-inoculated when receiving rigid and flexible implants. The final series of experiments compared IL-17A KO mice with WT C57BL/6 mice.

### Bacteria and inoculum preparation

The test microorganisms were *S. epidermidis* Epi 103.1 (CCOS 1152) and *S. aureus* JAR06.01.31 (CCOS 890), both obtained from human patients with implant-related bone infection. Both strains have a weak *in vitro* biofilm formation capacity (unpublished observations) according to the scoring system of Stepanovic et al. (Stepanovic et al., 2000). The inocula were prepared as previously described (Sabate Bresco et al., 2017b). The administered inoculum per animal was 1×10^4^ Colony Forming Units (CFU).

### Surgery

Commercially pure Titanium (CpTi) 4-hole rigid and CpTi 4-hole flexible MouseFix implants, and Titanium Aluminium Niobium alloy [Ti6Al4Nb (TAN)] screws (RISystem AG) were used in this study. Prior to use, all implants were cleaned and sterilized as previously described (Rochford et al., 2016).

Surgery was performed as previously described (Sabate Bresco et al., 2017b). Briefly, under general anesthesia with isoflurane (2–3% isoflurane in 100% O_2_, 1 l/min) (Isofluran Baxter^®^, Baxter AG), the subcutaneous Fascia lata was cut and the tissue plane between the quadriceps and the biceps femoris muscle was bluntly dissected. A Teflon foil was placed around the femur to protect the soft tissue from contamination. The implant was then fixed to the bone using four self-tapping, angular stable screws and then a 0.44 mm defect osteotomy was performed. In the inoculated groups, 2.5 µl of bacteria suspension (containing approximately 1×10^4^ CFU) was injected in the osteotomy site. The foil was then removed and the Fascia lata and the skin were closed with continuous sutures (5-0 Vicryl rapide, Ethicon). Analgesia and animal monitoring were performed as previously described. Weights and radiographs of the operated limb were taken weekly.

### Euthanasia and sample collection

At each scheduled time-point, the animals were euthanized by cervical dislocation after inducing general anesthesia in an induction box (5% isoflurane in 100% O_2_, 1 l/min). For quantitative bacteriology, the diaphysis of the operated femur, the implant, and the surrounding soft tissue were harvested separately in sterile containers with cold PBS. Bone and soft tissue samples were mechanically homogenized (Omni Tissue Homogenizer and Hard Tissue Homogenizing tips, Omni International). The implant was sonicated to remove adherent bacteria as described below. Moreover, aliquots of the bone homogenate were kept for cytokine/chemokine quantification or further processed for flow cytometry analysis as described below. Spleens and popliteal lymph nodes were harvested in cold PBS and complete RPMI (cRPMI: RPMI 1640, Gibco), penicillin (100 U/ml, Sigma-Aldrich), streptomycin (100 μg/ml, Sigma-Aldrich), kanamycin (0.1 μg/ml, Gibco), MEM vitamin (1x, Sigma-Aldrich), L-glutamine (2 mM, Sigma-Aldrich), sodium pyruvate (1 mM, Sigma-Aldrich), non-essential amino acids (1×, Sigma-Aldrich) and heat-inactivated fetal calf serum (10%, Sigma-Aldrich), respectively, and processed as previously described to obtain single cell suspensions for flow cytometry and cell culture (Rochford et al., 2016). For gene expression measurements, the diaphysis of the operated femur (including bone marrow and other tissues in the fracture site) were collected in RNAlater (Qiagen, Hilden, Germany).

### Cytokine/chemokine quantification

Bone homogenates stored at −80°C were thawed, centrifuged (9000 ***g***, 4°C, 10 min) and the supernatants collected for cytokine and chemokine quantification. Those were measured using a Milliplex MAP Mouse cytokine/chemokine Magnetic Bead Panel (Merck) for: G-CSF, IFN-γ, IL-2, IL-4, IL-6, IL-10, IL-13, IL-17A, KC, MCP-1 and TNF-α. Additionally, spleen single cell suspensions resuspended in cRPMI were plated in 24-well plates (1×10^6^ cells/well) and were stimulated with UV-killed *S. epidermidis* (at two, ten and 50 bacterial cells/splenocyte) and then incubated at 37°C, 5% CO_2_. Culture supernatants were collected 48 h later. Using Milliplex MAP Mouse cytokine/chemokine Magnetic Bead Panel (Merck) the following analytes were measured: IFN-γ, IL-2, IL-4, IL-10, IL-17A and TNF-α. Bone homogenates from the IL-17A study were centrifuged (9000 ***g***, 4°C, 10 min) and supernatants were collected and stored at −80°C until use. IFN-γ, IL-4, IL-6, IL-17A, IL-17F, IL-22, IL-33 and KC were then measured in those samples using a U-PLEX Biomarker Group 1 (mouse) assay (Meso Scale Discovery, Rockville, MD, USA), following the manufacturer's instructions. Samples were incubated in the plate at 4°C overnight to improve detection sensitivity.

### Myeloperoxidase analysis

Bone homogenates from day 3 post-surgery of C57BL/6 mice, previously frozen at −80°C, were thawed, centrifuged at 9000 ***g*** for 5 min at 4°C and the supernatant collected. Myeloperoxidase (MPO) levels were measured in those supernatants using a mouse MPO ELISA kit (Boster Biological Technology), according to the manufacturer's instructions.

### Flow cytometry

Samples for flow cytometry (bone homogenate and popliteal lymph node) were filtered through a 70 µm cell strainer (BD Biosciences) to obtain single cell suspensions and resuspended in cRPMI before staining. Bone single cell suspensions were stained with Fixable Viability Dye eFluor780 (eBioscience, San Diego, CA, USA) and for surface markers, as described in Table S5. For panels including cytokine secretion, cells were stimulated with PMA (50 ng/ml, Sigma-Aldrich), ionomycin (500 ng/ml, Sigma-Aldrich) and Brefeldin A (1×, eBioscience) for 4 h at 37°C, 5% CO_2_. Cells were subsequently stained with Fixable Dye eFluor780 and for surface markers before fixation and permeabilization using Intracellular Fixation & Permeabilization Buffer (eBioscience). Finally, samples were stained intracellularly for cytokines, using the antibodies listed in Table S5. All samples were analyzed using a Gallios flow cytometer (Beckman Coulter). Gating strategies are shown in Figs S1–S3.

### Quantitative bacteriology

Total viable bacterial counts were determined by plating bone homogenate or soft tissue homogenate on 5% horse blood agar (BA) (Oxoid). The number of bacteria associated with the implant was determined by sonicating the implant and screws in PBS for 3 min (Bandelin Sonorex at 40 kHz), vortex mixing for 10 s, and finally plating serial dilutions on BA. All BA plates were incubated at 37°C and CFU counts taken at 24 and 48 h. Mice were considered as infected when at least one sample (bone, soft tissue or implant) was culture positive. A representative colony recovered from each animal were confirmed to be *S. epidermidis* Epi 103.1 by Random Amplification of Polymorphic DNA (RAPD) (Versalovic et al., 1991).

### RNA extraction and gene expression evaluation

The femoral diaphysis from the Gene expression study groups were collected in RNAlater (Qiagen) and stored at −80°C after an initial period of 24 h at 4°C. RNA extraction was performed using a Tissue Lyse kit (Qiagen) following the manufacturer's instructions. RNA amount and quality were quantified using a NanoDrop 1000 Spectrophotometer (Thermo Fisher Scientific, Switzerland) and integrity was assessed using P200 Screen Tape (Agilent Technologies, Switzerland). All analyzed samples had an A230/A260 index greater than 2 and RIN values were greater than 6. Reverse transcription was performed on 400 ng of RNA/sample using Reaction Buffer (5x), dNTP Mix (10 mM), Random Hexamer Primer (0.2 µg/µl), RiboLock RNase Inhibitor (20 U/µl) and RevertAid Reverse Transcriptase (200 U/µl) (Thermo Fisher Scientific). mRNA expression of 44 genes was assessed using custom TaqMan^®^ Array Microfluidic cards (Affymetrix/Thermo Fisher Scientific), with 100 ng of cDNA loaded into each port (selected genes, representative of the bone healing process, tissue regeneration and immune responses, are listed in Table S4). mRNA expression in the Microfluidic cards was measured using a QuantStudio 7 Flex Real-Time PCR System (Thermo Fisher Scientific). To evaluate differences in gene transcription, the ΔΔCt method was used, with *18S*, *Eef2* and *Gapdh* as endogenous controls, and a control group (femurs of non-operated mice) was used as a calibrator unless indicated otherwise.

### Statistical analysis

Statistical analysis was conducted using GraphPad Prism 7 software (GraphPad Software). Statistical tests performed as indicated in figure legends. Differences were considered significant with *P*<0.05.

## Supplementary Material

Supplementary information
