# Supplementary material for: Fracture biomechanics influence local and systemic immune responses in a murine fracture-related infection model
Source: Biol Open. 2021 Oct 1;10(9):bio057315. doi: 10.1242/bio.057315 (PMC8496694; doi:10.1242/bio.057315)
Supplement: Supplementary information [file biolopen-10-057315-s1.pdf]

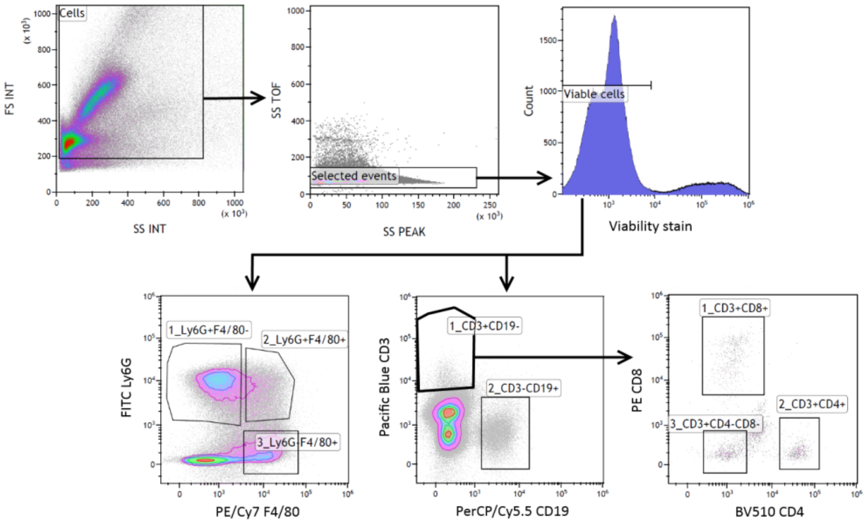

Fig. S1. Flow cytometry gating strategy of bone single cell suspensions. Cells were stained with viability dye and the antibodies listed in the Bone panel shown in supplementary Table 5. Upon selection of cells, doublets were excluded as well as dead cells before gating for the different surface markers.

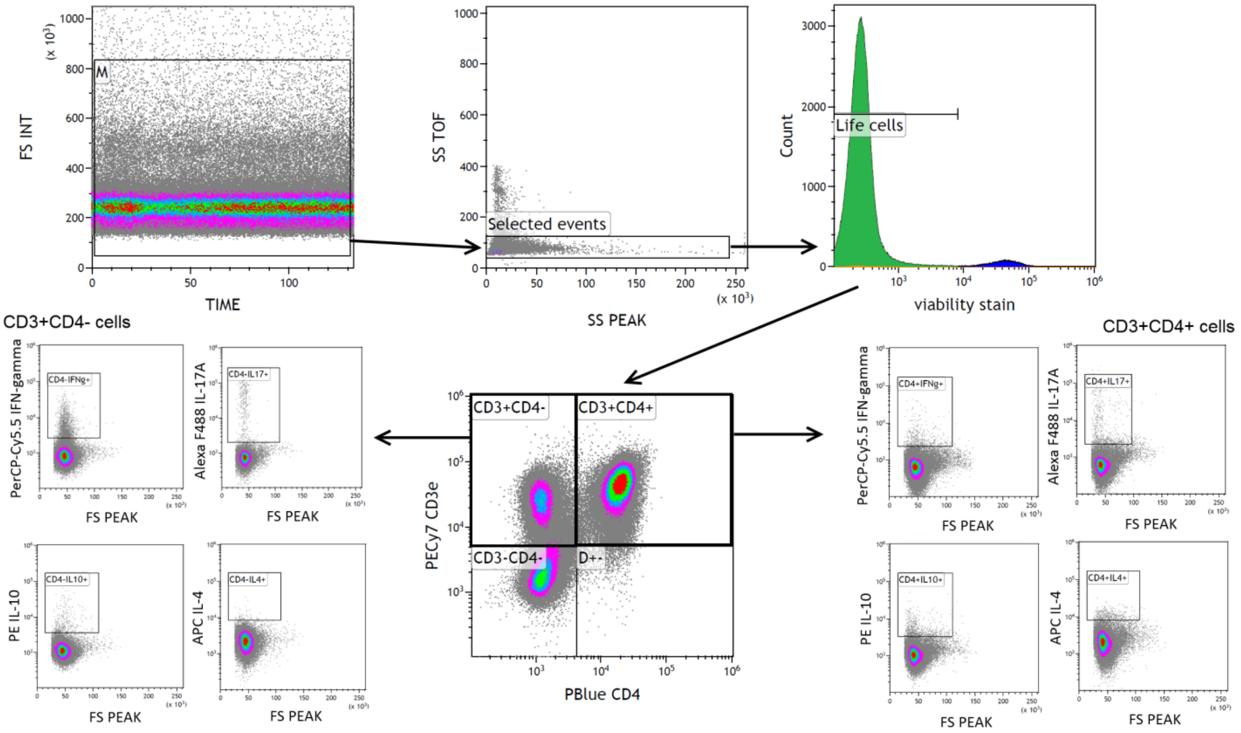

Fig. S2. Flow cytometry gating strategy of popliteal lymph node single cell suspensions. Cells were stained with viability dye and the antibodies listed in the Lymph Node panel shown in supplementary Table 5. Upon selection of cells, doublets and dead cell were excluded.

Biology Open • Supplementary information

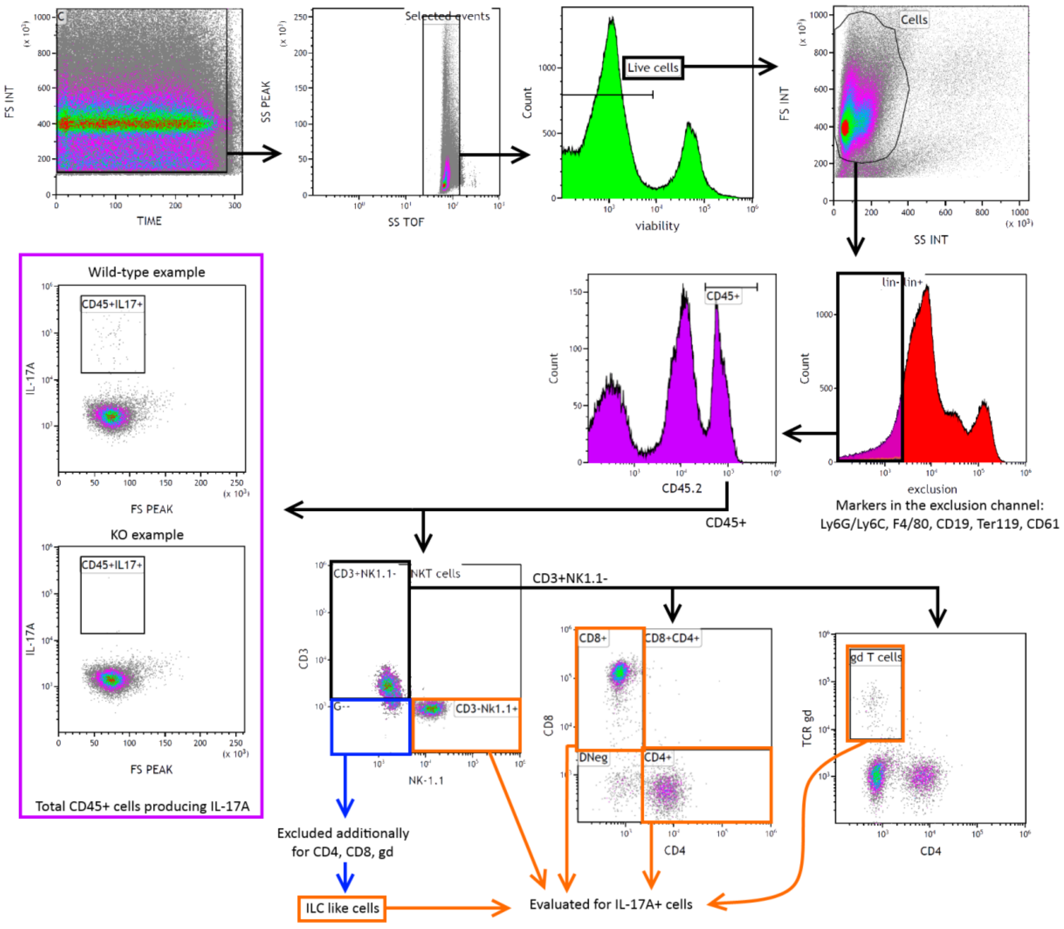

Fig. S3. Flow cytometry gating strategy of bone single cell suspensions. Cells were stained with viability dye and the antibodies listed in the IL-17A Bone panel shown in supplementary Table 5.

Biology Open • Supplementary information

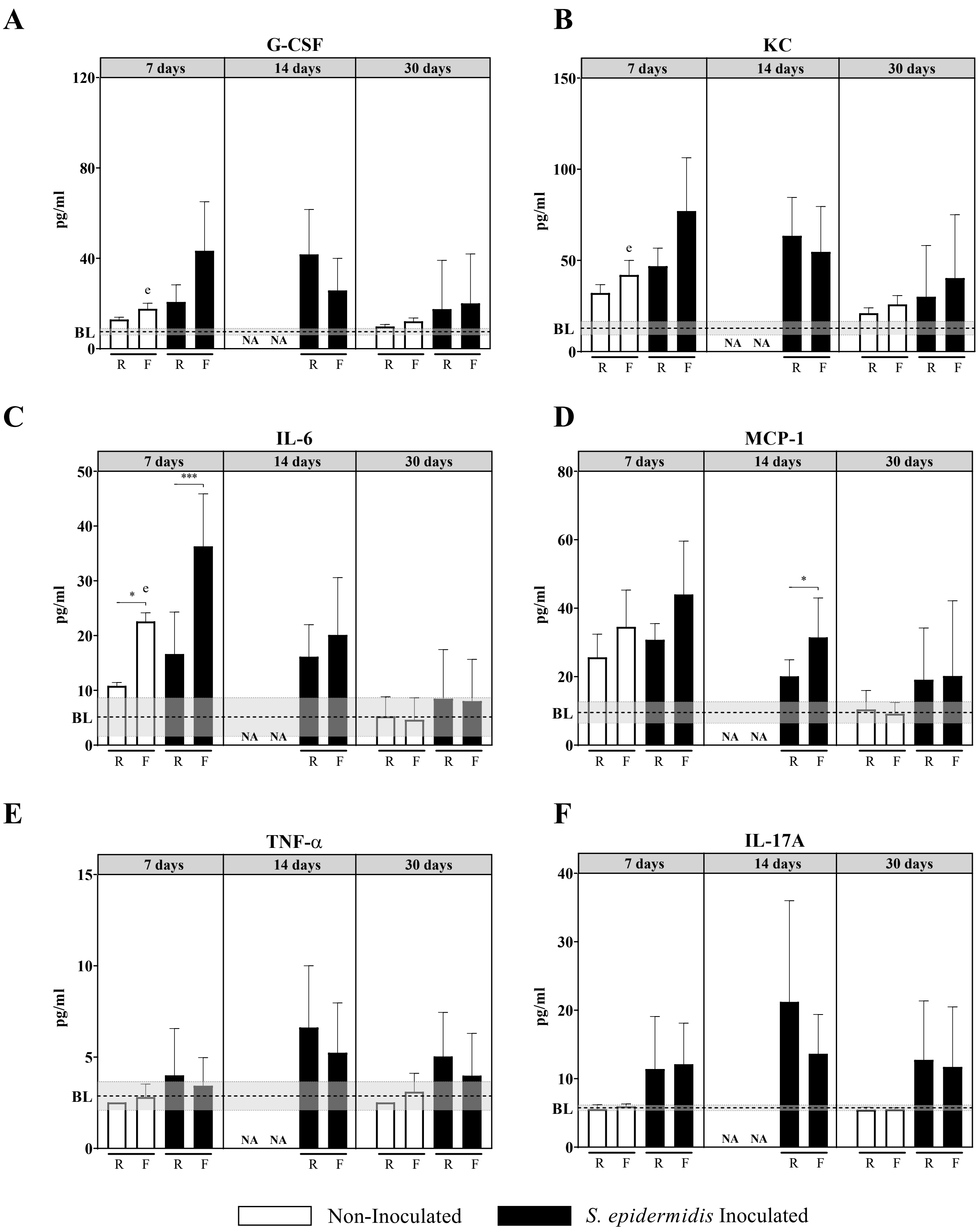

Fig. S4. Cytokine levels (pg/ml) in bone homogenate supernatants of BALB/c mice at 7, 14 and 30 days post-op. Data shown are Mean+SD (n=3-7). BL: baseline, mean of the control group (non-operated mice); grey area: BL ±SD of the control group. 2-way ANOVA per time point with Tukey post-hoc correction. Statistics summarize significant differences in the following comparisons: e) Non-inoculated vs *S. epidermidis* Inoculated; Rigid vs Flexible implant within each condition: * P<0.05; ** P<0.01, ***P< 0.001. R: rigid implant; F: flexible implant, NA: Not available.

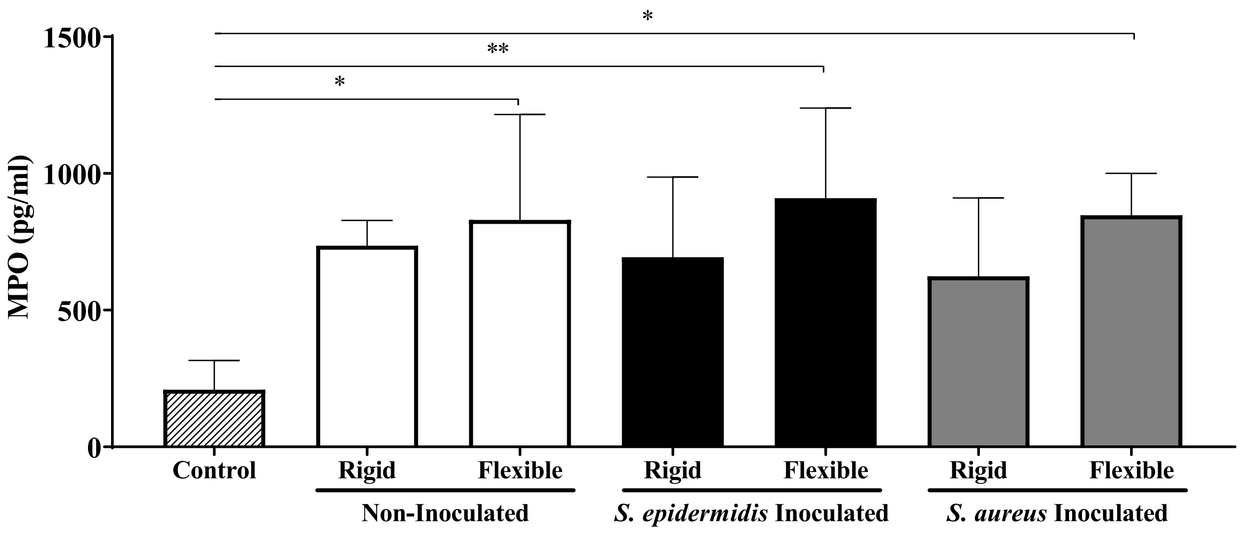

Fig. S5. MPO levels at day 3 post-op in bone homogenate supernatants. Mean values and SD (n=4-7). Control: contralateral femurs. 2-way ANOVA with Sidak post-hoc for comparison between different conditions (type of implant and infection status), Kruskal-Wallis test with Dunn's post-hoc for comparison of all conditions with the control group (non-operated): * $P<0.05$; ** $P<0.01$.

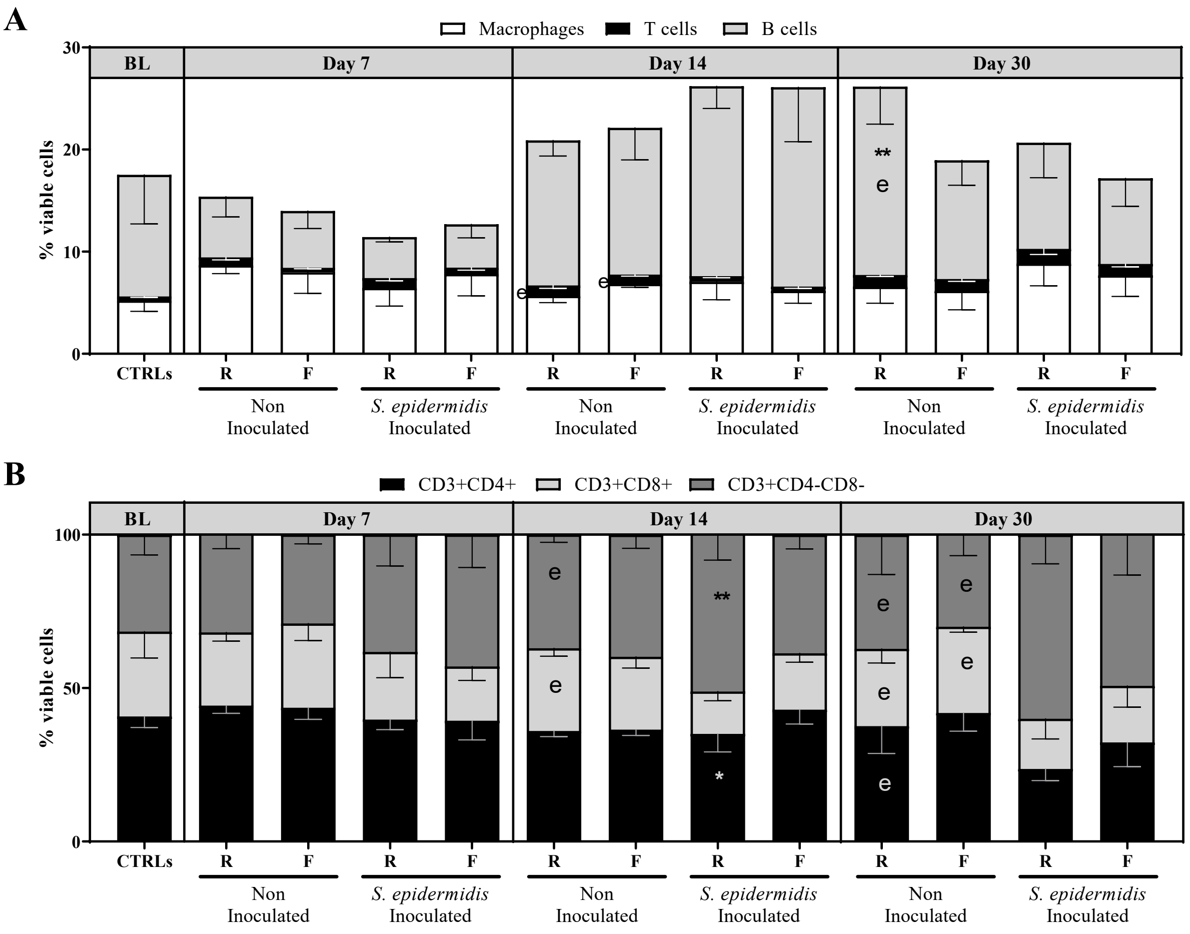

Fig. S6. Macrophage, T cell and B cell populations observed over time at the fracture site in Balb/C mice. Upper panel, macrophage lineage cells (Ly6G-F4/80+), T cells (CD3+CD19-) and B lineage cells (CD19+CD3-) as a percentage of total viable cells (A). Lower panel, percentage of CD4+, CD8+ and CD4-CD8- calculated on CD3+ cell numbers (B), in bone single cell suspensions of Balb/C mice at days 7, 14 and 30 post-operatively. Mean values and SD (n=3-8). 2-way ANOVA per time point with Tukey post-hoc correction. Statistics summarize significant differences in the following comparisons: Non-inoculated vs *S. epidermidis* denoted by e); Rigid vs Flexible implant within each condition: * *P<0.05; *** *P<0.01,* ****P< 0.001*. BL: baseline, mean of the control group (non-operated). R: rigid implant; F: flexible implant.

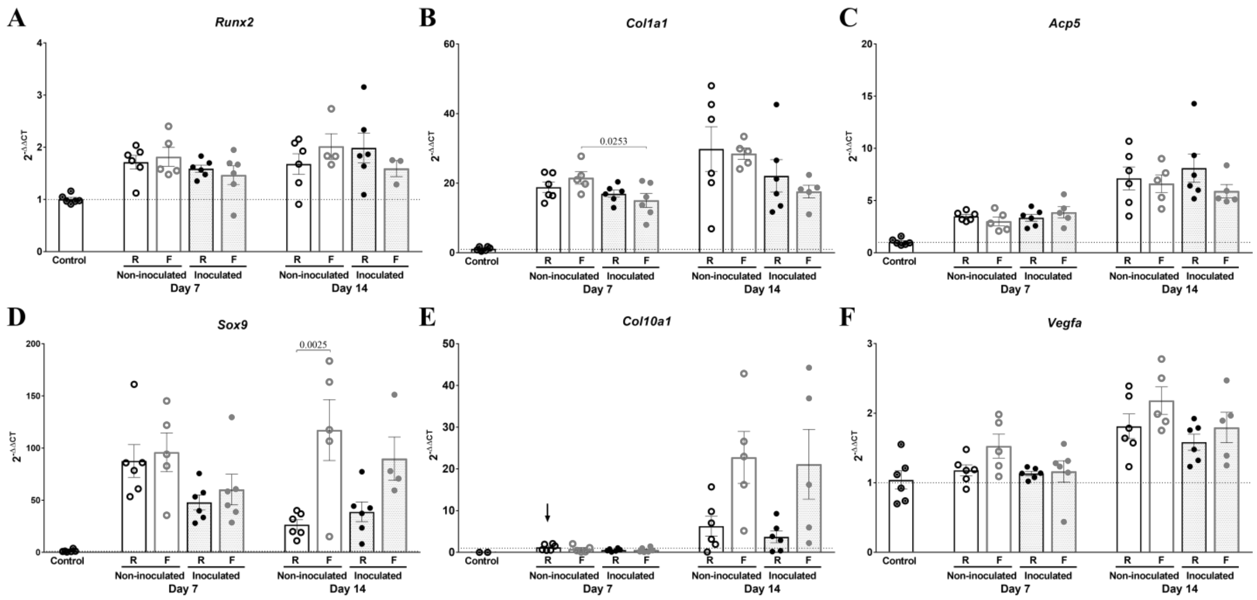

Fig. S7. mRNA expression in bones of C57BL/6 mice at 7 and 14 days post-op. $2^{-\Delta\Delta CT}$ of *Arg1*, *Nos2*, *Ccl2*, *Cd80*, *Il33* and *Hif1a* in RNA isolated from operated and not operated femurs; at 0, 7 and 14 days post op. *18S*, *Eef2* and *Gapdh* used as endogenous controls, control group used as reference group (calibrator). Mean values and SD (n=5-6). 2-way ANOVA per time point, with Sidak's post-hoc test, P-values < 0.05 depicted in figure. R: rigid implant; F: flexible implant.

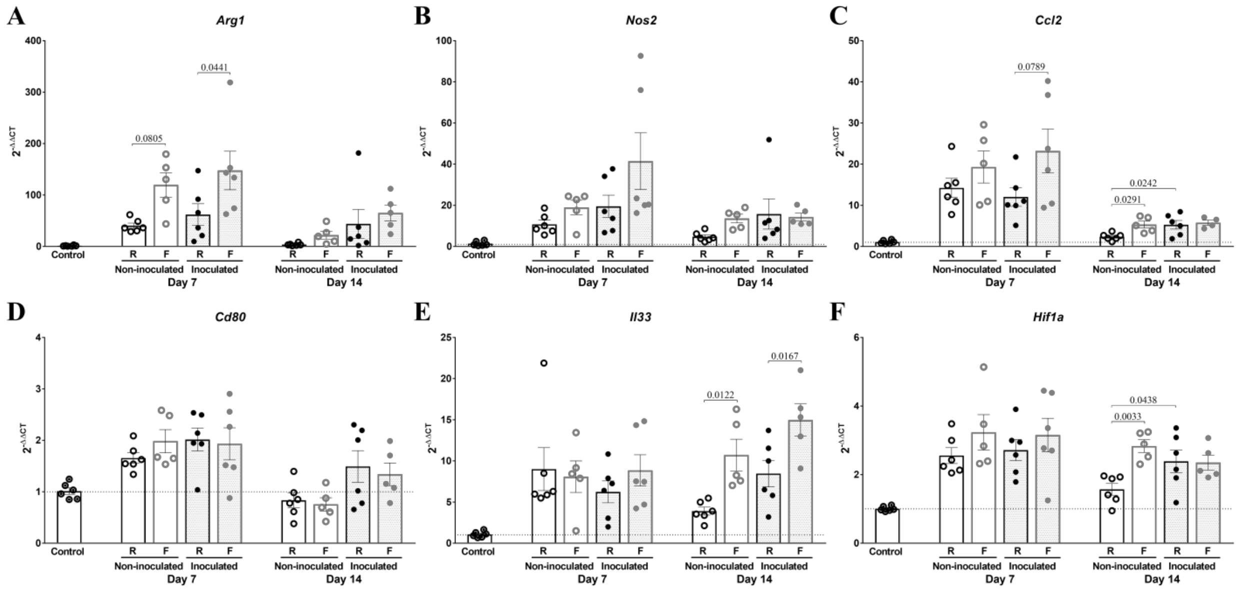

Fig. S8. mRNA expression in bones of C57BL/6 mice at 7 and 14 days post-op. $2^{-\Delta\Delta CT}$ of *Runx2, Col1a1, Acp5, Sox9, Col10a1* and *Vegfa* in RNA isolated from operated and not operated femurs; at 0, 7 and 14 days post op. *18S*, *Eef2* and *Gapdh* used as endogenous controls, control group used as reference group (calibrator) except for *Col10a1* where rigid non-inoculated group at day 7 was used as reference due to almost no gene expression in control animals (black arrow). Mean values and SD (n=5-6). 2-way ANOVA per time point, with Sidak's post-hoc test, P-values <0.05 depicted in figure. R: rigid implant; F: flexible implant.

Biology Open • Supplementary information

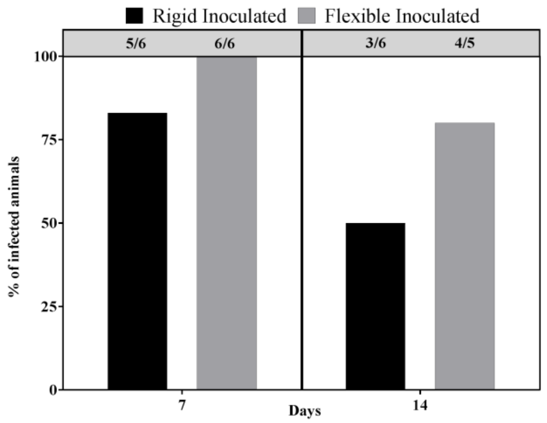

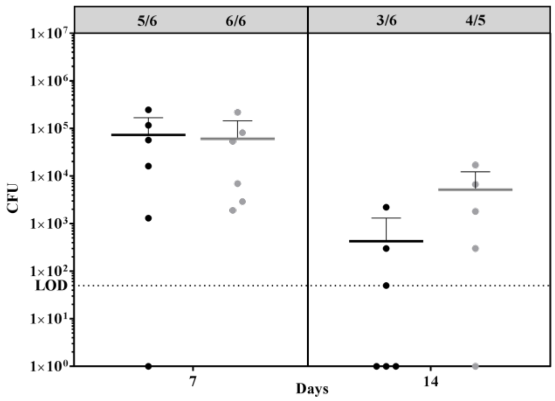

Fig. S9. Percentage of C57BL/6 mice infected and CFU counts at days 7 and 14 post-op. Percentage of infected mice (left), and total CFU counts (sum of CFU from soft tissue and implant) (right). Number of culture-positive animals/total number of animals per groups are shown in the upper panel of each graph. Mean values and SD (n=5-6). LOD: Limit of detection, $0.5x10^2$, culture negative samples are represented as 1. Fisher's exact test and Mann-Whitney test. No significant differences observed.

Biology Open • Supplementary information

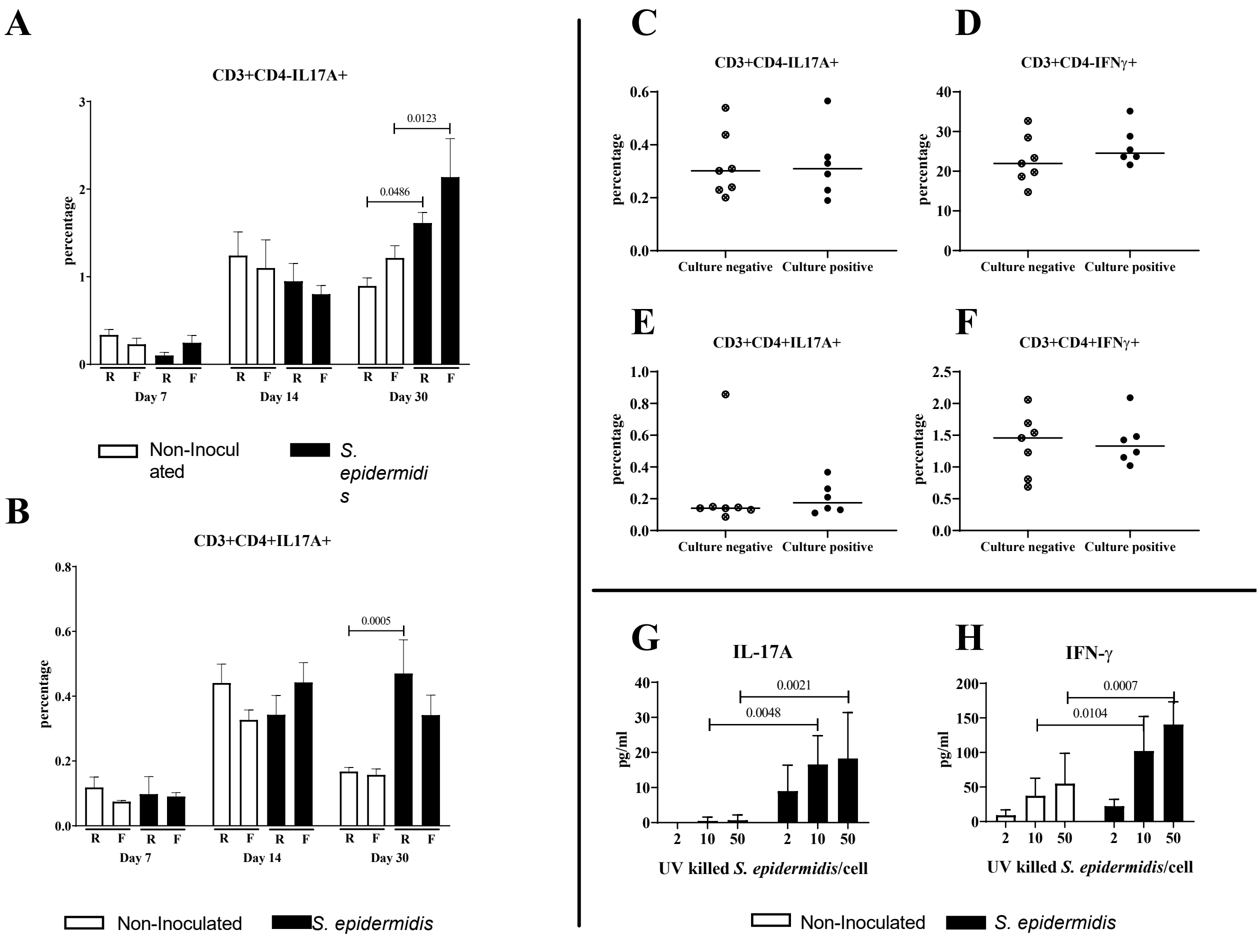

Fig. S10. Systemic immune responses associated with infection in Balb/C mice. A-B) Percentages of CD3+CD4-IL-17A+ and CD3+CD4+IL-17A+ T cells in popliteal lymph node single cell suspensions, for both rigid (R) and flexible (F) plate groups. Data shown are Mean+SD (n=4-8). Two-way ANOVA with Tukey post-hoc correction per time-point. C-F) Percentage of IL-17A+ and IFN-γ+ T lymphocytes in popliteal lymph nodes at day 14 in culture-negative mice (infection cleared) or culture-positive mice (infected). Both rigid and flexible samples grouped together (n=6-7). Mann-Whitney test. G) IL-17A and H) IFN-γ production by splenocytes from non-inoculated and *S. epidermidis* inoculated BALB/c mice at day 30 (rigid and flexible samples grouped together) after stimulation with UV-killed *S. epidermidis in vitro* (dose indicated in the x-axis: ratio of bacteria per spleen cell). Data shown are Mean+SD (n=5-6). 2-way ANOVA with Sidak post-hoc correction.

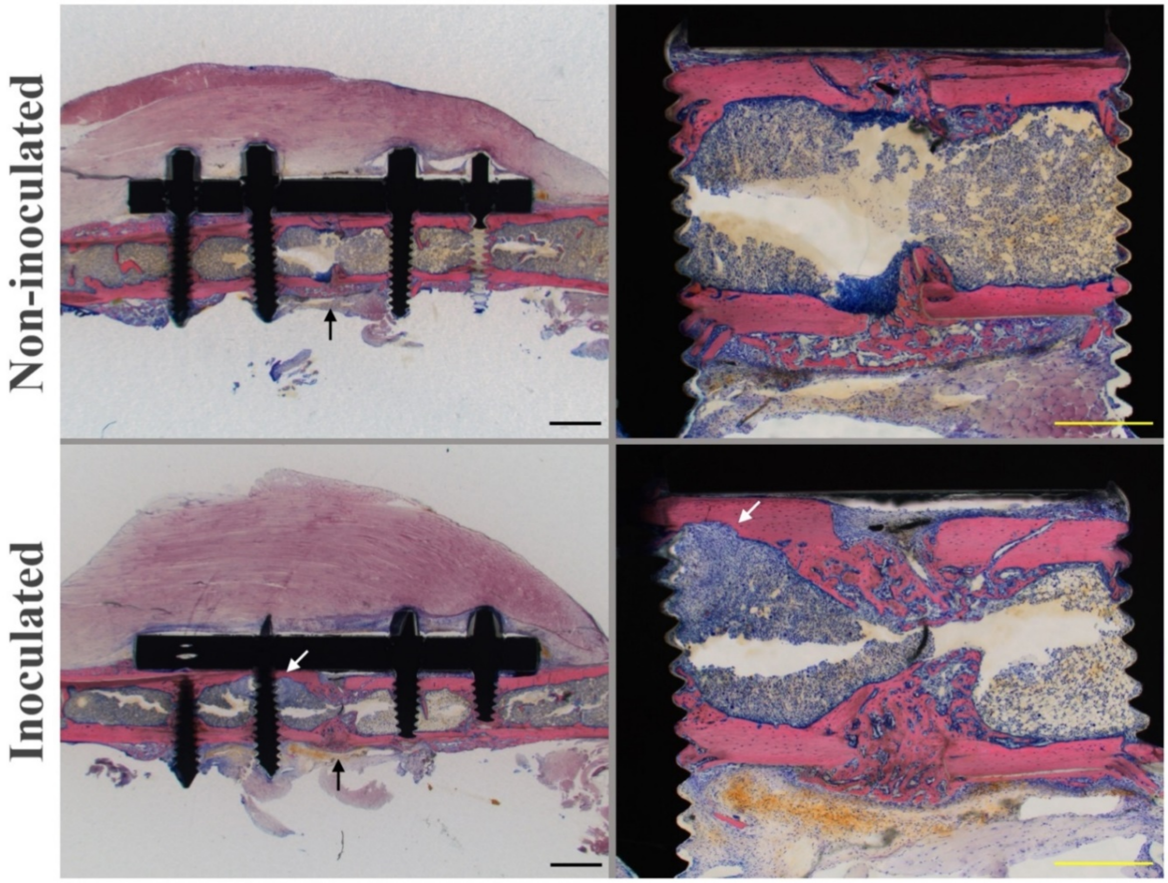

Fig. S11. Light microscopic images of Giemsa/Eosin stained MMA sections of C57BL/6 IL-17A KO mice non-inoculated and *S. epidermidis* inoculated at day 14. Scale bar overview images: 1000 µm. Scale osteotomy magnification: 500 µm. The osteotomy gap was filled with new bone in both groups (black arrows). *S. epidermidis* inoculated mice only showed localized signs of infection as osteolytic regions around the screws with granulocyte infiltrate (white arrows). Similar observations were done when comparing with WT non-inoculated and *S. epidermidis* inoculated from previous data (Sabaté Brescó et al., 2017b). Histology sections were generated as previously described (Sabate Brescó et al., 2017b)

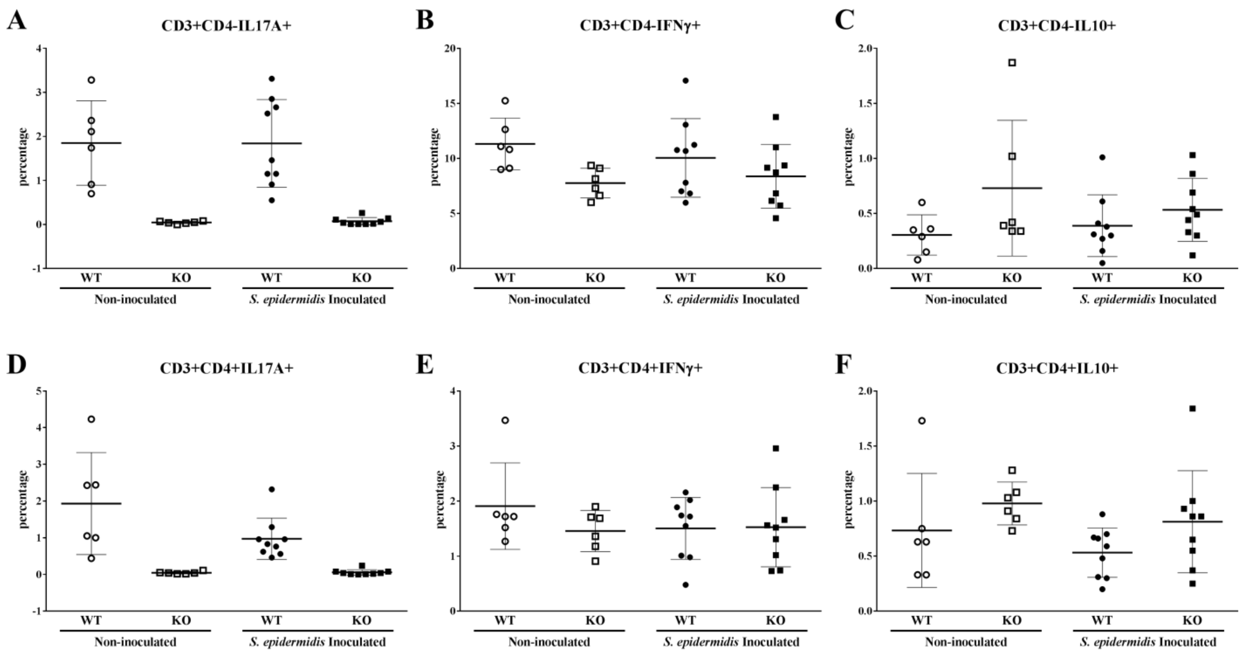

Fig. S12. Percentage of IL-17A, IFN-γ and IL-10 producing T cells in popliteal lymph node of non-inoculated and *S. epidermidis* inoculated C57BL/6 WT and C57BL/6 IL17A KO mice with a rigid implant, 14 days post-op. Data shown are Mean+SD (n=7-11). 1-way ANOVA with Sidak's post-hoc test or Kruskal-Wallis test with Dunn's post hoc test (no statistics performed for IL-17A populations between WT and KO).

Biology Open • Supplementary information

Table S1. Study design of the initial study, from which samples were obtained in order to characterize immune responses.

	Day 3		Day 7		Day 14		Day 30	
**Group**	**C57BL/6**	**BALB/c**	**C57BL/6**	**BALB/c**	**C57BL/6**	**BALB/c**	**C57BL/6**	**BALB/c**
Non-inoculated								
Rigid	≥ 6	NA	≥ 6	4	≥ 6	4	≥ 6	≥ 6
Flexible	≥ 6	NA	≥ 6	≥ 6	≥ 6	3	≥ 6	≥ 6
S. epidermidis Inoculated								
Rigid	≥ 6	NA	≥ 6	4	≥ 6	≥ 6	≥ 6	≥ 6
Flexible	≥ 6	NA	5	≥ 6	≥ 6	≥ 6	≥ 6	≥ 6
S. aureus Inoculated								
Rigid	≥ 6	NA	≥ 6	NA	≥ 6	NA	-	-
Flexible	≥ 6	NA	5	NA	≥ 6	NA	-	-

NA: Not applicable

Table S2. Gene expression study design. Final number of animals per group.

**Gene expression study groups (C57BL/6 mice)**					
	**Day 0**	**Day 7**		**Day 14**	
**Group**	**Non-operated**	**Rigid**	**Flexible**	**Rigid**	**Flexible**
Non-inoculated	6	6	5	6	5
S. epidermidis inoculated	-	6	6	6	5

Table S3. IL-17A study design. Final number of animals per group.

**IL-17A study groups (C57BL/6 mice)**					
	**Day 0**	**Day 14**		**Day 30**	
**Group**	**Non-operated**	**Non-inoculated**	***S. epidermidis* inoculated**	**Non-inoculated**	***S. epidermidis* inoculated**
Wild type	4	9	8	-	9
IL-17A KO	3	9	8	-	7

Biology Open • Supplementary information

Table S4. List of genes included in the microfluidic card

#	Gene name* (common abbreviation)	Gene symbol*	Gene ID*	Applied Biosystems® Assay ID	Amplicon Length
1	Adhesion G protein-coupled receptor E1 (F4/80)	*Adgre1*	13733	Mm00802529_m1	92
2	Mannose receptor, C type 1 (CD206)	*Mrc1*	17533	Mm00485148_m1	76
3	Arginase, liver (Arg-1)	*Arg1*	11846	Mm00475988_m1	65
4	Nitricoxidesynthase 2, inducible (iNos-2)	*Nos2*	18126	Mm00440502_m1	66
5	Elastasa, neutrophilexpressed	*Elane*	50701	Mm01168928_g1	69
6	Tumor necrosis factor (ligand) superfamily, member 11 (RANKL)	*Tnfsf11*	21943	Mm00441906_m1	66
7	CD80 antigen	*Cd80*	12519	Mm00711660_m1	117
8	Tumor necrosis factor (TNF-α)	*Tnf*	21926	Mm00443258_m1	81
9	Interleukin 4 (IL-4)	*Il4*	16189	Mm00445259_m1	79
10	Interleukin 6 (IL-6)	*Il6*	16193	Mm00446190_m1	78
11	Interleukin 10 (IL-10)	*Il10*	16153	Mm00439614_m1	79
12	Interleukin 17A (IL-17A)	*Il17a*	16171	Mm00439618_m1	80
13	Interleukin 17F (IL-17F)	*Il17f*	257630	Mm00521423_m1	85
14	Interleukin 23, alpha subunit p19 (IL-23)	*Il23a*	83430	Mm01160011_g1	109
15	transforming growth factor, beta 1 (TGF-beta1)	*Tgfb1*	21803	Mm01178820_m1	59
16	transforming growth factor, beta 2 (TGF-beta2)	*Tgfb2*	21808	Mm00436955_m1	82
17	transforming growth factor, beta 3 (TGF-beta3)	*Tgfb3*	21809	Mm00436960_m1	60
18	colony stimulating factor 1, macrophage (M-CSF)	*Csf1*	12977	Mm00432686_m1	70
19	colony stimulating factor 3, granulocyte (G-CSF)	*Csf3*	12985	Mm00438335_g1	63
20	colony stimulating factor 2, granulocyte-macrophage (GM-CSF)	*Csf2*	12981	Mm01290062_m1	125
21	Interleukin 33 (IL-33)	*Il33*	77125	Mm00505403_m1	83
22	Chemokine (C-C motif) ligand 2 (CCL2)	*Ccl2*	20296	Mm00441242_m1	74
23	chemokine (C-C motif) receptor 2 (CCR2)	*Ccr2*	12772	Mm01216173_m1	88
24	Toll-like receptor 2 (TLR-2)	*Tlr2*	24088	Mm00442346_m1	69
25	Toll-like receptor 4 (TLR-4)	*Tlr4*	21898	Mm00445273_m1	87
26	Vascular endothelial growth factor A (VEGF-A)	*Vegfa*	22339	Mm01281449_m1	81
27	Hypoxia inducible factor 1, alpha subunit (HIF1- α)	*Hif1a*	15251	Mm00468869_m1	75
28	Endothelial PAS domain protein 1 (HIF2-α)	*Epas1*	13819	Mm01236112_m1	63
29	S100 calcium binding protein A8 (S100A8)	*S100a8*	20201	Mm00496696_g1	131
30	S100 calcium binding protein A9 (S100A9)	*S100a9*	20202	Mm00656925_m1	162
31	High mobility group box 1 (HMGB1)	*Hmgb1*	15289	Mm00849805_gH	158
32	Caspase 3	*Casp3*	12367	Mm01195084_m1	79
33	Selectin, lymphocyte (CD62L)	*Sell*	20343	Mm00441291_m1	101
34	Tumor necrosis factor receptor superfamily, member 11b (osteoprotegerin, OPG)	*Tnfrsf11b*	18383	Mm01205928_m1	75
35	Secretedphosphoprotein 1 (OPN)	*Spp1*	20750	Mm00436767_m1	114
36	Collagen, type I, alpha 1 (ColIα1)	*Col1a1*	12842	Mm00801666_g1	89
37	Collagen, type X, alpha 1 (ColXα1)	*Col10a1*	12813	Mm00487041_m1	77
38	Bridgingintegrator 1 (ALP-1)	*Bin1*	30948	Mm00437457_m1	72
39	Runt related transcription factor 2 (Runx2)	*Runx2*	12393	Mm00501584_m1	91
40	Acidphosphatase 5, tartrateresistant (TRAP)	*Acp5*	11433	Mm00475698_m1	79
41	SRY (sex determining region Y)-box 9 (Sox9)	*Sox9*	20682	Mm00448840_m1	101
42	Eukaryotic translation elongation factor 2 (eEF-2)	*Eef2*	13629	Mm01171434_g1	74
43	Glyceraldehyde-3-phosphate dehydrogenase (GAPDH)	*Gapdh*	14433	Mm99999915_g1	109
44	Ribosomal 18S	*18s*	-	-	-

** Details according to NCBI*

Table S5. Flow cytometry panels details.

Product	Clone (If applicable)	Company
Fixable Viability Dye eFluor780	NA	eBioscience
**Bone panel**		
FITC anti-mouse Ly6G Antibody	1A8	Biolegend
PE anti-mouse CD8a Antibody	53-6.7	Biolegend
PerCP/Cy5.5 anti-mouse CD19 Antibody	6D5	Biolegend
PE/Cy7 anti-mouse F4/80 Antibody	BM8	Biolegend
APC anti-mouse CD138 (Syndecan-1) Antibody	281-2	Biolegend
Pacific Blue anti-mouse CD3ε Antibody	145-2C11	Biolegend
Brilliant Violet 510 anti-mouse CD4 Antibody	RM4-5	Biolegend
**General lymph node panel**		
PE/Cy7 anti-mouse CD3ε Antibody	145-2C11	Biolegend
Pacific Blue anti-mouse CD4 Antibody	RM4-5	Biolegend
Alexa Fluor 488 anti-mouse/rat IL-17A Antibody	eBio17B7	eBioscience
PE anti-mouse IL-10 Antibody	JES5-16E3	eBioscience
PerCP-Cy5.5 anti-mouse IFN-gamma Antibody	XMG1.2	eBioscience
APC anti-mouse IL-4 Antibody	11B11	eBioscience
**IL-17A Bone panel**		
PE anti-mouse CD19 Antibody	6D5	Biolegend
PE anti-mouse TER-119/Erythroid Cells Antibody	TER-119	Biolegend
PE anti-mouse Ly-6G/Ly-6C (Gr-1) Antibody	RB6-8C5	Biolegend
PE anti-mouse F4/80 Antibody	BM8	Biolegend
PE anti-mouse/rat CD61 Antibody	2C9.G2 (HMß3-1)	Biolegend
Alexa Fluor® 700 anti-mouse CD45 Antibody	30-F11	Biolegend
PerCP/Cy5.5 anti-mouse NK-1.1 Antibody	PK136	Biolegend
PE/Cy7 anti-mouse CD8a Antibody	53-6.7	Biolegend
APC Anti-mouse TCR γ/δ Antibody	GL3	Biolegend
Pacific Blue anti-mouse CD3ε Antibody	145-2C11	Biolegend
Brilliant Violet 510 anti-mouse CD4 Antibody	RM4-5	Biolegend
Alexa Fluor 488 anti-mouse/rat IL-17A Antibody	eBio17B7	eBioscience

Biology Open • Supplementary information
